# Supplementary material for: Awake alpha bursting emerges as the dynamic working state in a lateral geniculate thalamocortical cell model
Source: PLoS Comput Biol. 2026 Oct 8;22(10):e1014654. doi: 10.1371/journal.pcbi.1014654 (PMC13649143; doi:10.1371/journal.pcbi.1014654)
Supplement: Supplementary file 1 [file pcbi.1014654.s001.pdf]

Supplemental Methods

Differential equations, kinetic rate constants, ionic current equations and all associated parameter values for the computational TC cell are provided here. The equations representing the ionic current kinetics are taken from a variety of different modeling studies that can each be traced back to experimentally fit kinetic equations. An example modeling study using the given gated currents can be found at the following: I_{Na} [1], I_K [1], I_{Nap} [2], I_{CaT} [3] I_{CaL} [1], I_h [3], and I_M [1]. These modeling studies base their kinetic equations from the following experimental works: I_{Na} [4], I_K [4], I_{Nap} [5], I_{CaT} [6] I_{CaL} [7], I_h [6], and I_M [8]. All parameter values provided within these methods (including baseline excitation $I_{app} = 2.7\mu A/cm^2$) are set to produce high-threshold bursting at an alpha frequency. External excitation (or different ionic conductances) can then be varied to produce the other firing modes as desired/described in the main text.

The following differential equation describes a conductance based model where the change in membrane potential (V) of the TC cell is a combination of the ionic currents ΣI_{ionic} and the applied current I_{app} all scaled by 1 over the membrane capacitance C_M (in units $\mu F/cm^2$):

$$\frac{dV}{dt} = \frac{1}{C_M}(I_{app} - \Sigma I_{ionic}). \tag{1}$$

The summed ionic currents are:

$$\Sigma I_{ionic} = I_{Na} + I_K + I_l + I_{kl} + I_{Nap} + I_{CaT} + I_{CaL} + I_h + I_M. \tag{2}$$

These currents are: spiking sodium I_{Na}, spiking potassium I_K, standard leak I_l, potassium leak I_{kl}, persistent sodium I_{Nap}, T-type calcium I_{CaT}, L-type calcium I_{CaL}, hyperpolarization activated H current I_h, and non-inactivating slowly decaying potassium M current I_M. The kinetics for these currents are adapted from previously published studies (see above) and are reproduced here for the readers. Each current type j adheres to the Hodgkin-Huxley formulation:

$$I_j = g_j m_j^x h_j^y (V - E_j), \tag{3}$$

where g_j is the maximal conductance for current type j, there are x and y numbers of activation m and inactivation h gates respectively, and the current has reversal potential E_j. The kinetics of each ionic gating variable have been fit based on voltage clamp experiments and follow one of two identical (a transformation from one to the other exists [9]) schemes. These schemes are provided here for an arbitrary gating variable m:

$$\frac{dm}{dt} = (m_{inf} - m)/\tau_m \tag{4}$$

$$\frac{dm}{dt} = \alpha_m(1 - m) - \beta_m m \tag{5}$$

where the kinetics are described either by the steady state open probability m_{inf} and time constant functions τ_m, or via opening (α_m) and closing (β_m) kinetic rates. Using these gating equation formulations, the chosen modeled ionic currents are:

$$I_{Na} = g_{Na} m_{Na}^3 h_{Na}(V - E_{Na}) \tag{6}$$

$$I_K = g_K m_K^4 (V - E_K) \tag{7}$$

$$I_L = g_L(V - E_L) \tag{8}$$

$$I_{KL} = g_{KL}(V - E_{KL}) \tag{9}$$

$$I_H = g_H r(V - E_H) \tag{10}$$

$$I_{CAT} = g_{TLT} m_{TLT}^2 h_{TLT}(V - E_{CA}) \tag{11}$$

$$I_{CAL} = g_{cal} q_{cal}^2 r_{cal}(V - E_{CA}) \tag{12}$$

$$I_M = g_M p(V - E_K) \tag{13}$$

$$I_{NAP} = g_{NAP} m_{NAPinf}(V - E_{Na}). \tag{14}$$

(15)

Gating variables for the ionic currents are listed below:

Current	Activation Open Prob	Activation time constant	Inactivation Open Prob	Inactivation time constant
I_{Na}	$\frac{\alpha_m}{\alpha_m+\beta_m}$	$\frac{1}{\alpha_m+\beta_m}$	$\frac{\alpha_h}{\alpha_h+\beta_h}$	$\frac{\alpha_h}{\alpha_h+\beta_h}$
I_K	$\frac{\alpha_K}{\alpha_K+\beta_k}$	$\frac{1}{\alpha_K+\beta_k}$	NA	NA
I_L	NA	NA	NA	NA
I_{KL}	Na	NA	NA	NA
I_H	$\frac{1}{1+e^{((V+75)/5.5)}}$	$\frac{1}{e^{(-.086V-14.59)}+e^{(.07V-1.87)}}$	NA	NA
I_{CAT}	$\frac{1}{1+e^{(\frac{-(V+62)}{6.2})}}$	$.612+\frac{1}{e^{(\frac{-(V+135)}{16.7})}+e^{(\frac{(V+19.8)}{18.2})}}$	$\frac{1}{1+e^{((V+86)/4)}}$	$30.8+\frac{211.4+e^{((V+115.2)/5)}}{1+e^{((V+86)/3.2)}}$
I_{CAL}	$\frac{\alpha_q}{\alpha_q+\beta_q}$	$\frac{1}{\alpha_q+\beta_q}$	$\frac{\alpha_r}{\alpha_r+\beta_r}$	$\frac{1}{\alpha_r+\beta_r}$
I_M	$\frac{1}{1+e^{(-(V+35)/10)}}$	$\frac{1000}{3.3e^{((V+35)/20)}+e^{(-(V+35)/20)}}$	NA	NA
I_{NAP}	$\frac{1}{1+e^{(-(V+50)/5)}}$	NA	NA	NA

Table A: Open probability and time constant functional forms for both the activation and inactivation gating variables.

For instances where the open probability and the time constants are given in terms of opening (α) and closing (β) rates, these rate constants are listed below. Here Vt is a voltage shift (in mV) defined to as $Vt = V + 55$.

Current	Activation α	Activation β	Inactivation α	Inactivation β
I_{Na}	$\frac{0.32(13-Vt)}{e^{((13-Vt)/4)}-1}$	$\frac{0.28(Vt-40)}{e^{((Vt-40)/5)}-1}$	$0.128e^{((17-Vt)/18)}$	$\frac{4}{1+e^{((40V-Vt)/5)}}$
I_K	$\frac{0.032(15-Vt)}{e^{((15-Vt)/5)}-1}$	$0.5e^{((10-Vt)/40)}$	NA	NA
I_{CAL}	$\frac{.055(-27-V)}{e^{((-27-V)/3.8)}-1}$	$.94e^{((-75-V)/17)}$	$.000457e^{((-13-V)/50)}$	$\frac{.0065}{e^{((-15-V)/28)}+1}$

Table B: Rate constants for gating variables that are defined in terms of opening and closing rates.

Finally, the maximal conductances and reversal potentials for the 9 membrane bound ionic currents and the 1 synaptic current (described in detail in the main text) are provided below:

Current I_j	Maximal Conductance g_j (in mS/cm^2)	Reversal Potential E_j (in mV)
I_{Na}	90	$E_{na} = 50$
I_K	4.4	$E_k = -100$
I_L	.0195	$E_l = -85$
I_{KL}	.012	$E_k = -100$
I_H	.06	$E_h = -43$
I_{CAT}	4.04	$E_{Ca} = RT/(zF)ln(Ca_e/Ca_i)$
I_{CAL}	.222	$E_{Ca} = RT/(zF)ln(Ca_e/Ca_i)$
I_M	.12	$E_k = -100$
I_{NAP}	.013	$E_{na} = 50$
I_{syn}	.5	Excitatory: $E_{syn} = 0$, Inhibitory:$E_{syn} = -80$

Table C: An initial set of maximal conductance values that produces high-threshold bursting and the fixed reversal potentials for all of incoming currents. Note, for all bifurcation diagrams plotting I_{app} vs g_{kl} (or g_M), the maximal conductances are divided by .06 and .3 respectively for numerical purposes.

For the two calcium currents, I_{CaL} and I_{CaT}, the calcium flux and the corresponding reversal potential are explicitly time dependent and not just given as fixed parameter values. Notably E_{Ca} varies according to the dynamic variable of internal Calcium Ca_i whose changes are described by the ODE taken from [3] reproduced here:

$$\frac{dCa_i}{dt} = ((-I_{CaL} - I_{Ca_T})/(zFw)) - (Ca_i - Ca_r)/\tau_{Ca}. \tag{16}$$

All remaining parameters with their physiological meaning and value are given below:

Parameter	Description	Value
T	Temperature	$310.35K$
R	Gas Constant	8.314 J/molK
z	Ca++ ion valence	2
F	Faraday Constant	96485.3 C/mol
Ca_e	external calcium concentration	2mM
w	perimembrane shell thickness	$.5\mu m$
τ_{Ca}	Ca++ removal rate	10ms
Ca_r	resting Ca++ concentration	.00005mM
C_m	Capacitance	$1\mu F/cm^2$
τ_d	Synaptic decay constant	Excitatory: .7ms, Inhibitory: 5ms
τ_r	Synaptic Rise constant	Excitatory: .125ms, Inhibitory: .25ms
V_{max}	Synaptic cell max membrane potential	10mV

Table D: Additional fixed parameter values.

References

[1] Martin Pospischil, Maria Toledo-Rodriguez, Cyril Monier, Zuzanna Piwkowska, Thierry Bal, Yves Frégnac, Henry Markram, and Alain Destexhe. Minimal hodgkin–huxley type models for different classes of cortical and thalamic neurons. *Biological cybernetics*, 99:427–441, 2008.

[2] Guoshi Li and Thomas A Cleland. A two-layer biophysical model of cholinergic neuromodulation in olfactory bulb. *Journal of Neuroscience*, 33(7):3037–3058, 2013.

[3] Guoshi Li, Craig S Henriquez, and Flavio Fröhlich. Unified thalamic model generates multiple distinct oscillations with state-dependent entrainment by stimulation. *PLoS computational biology*, 13(10):e1005797, 2017.

[4] Roger D Traub and Richard Miles. *Neuronal networks of the hippocampus*. Cambridge University Press, 1991.

[5] CR French, P Sah, KJ Buckett, and PW Gage. A voltage-dependent persistent sodium current in mammalian hippocampal neurons. *The Journal of General Physiology*, 95(6):1139–1157, 1990.

[6] John R Huguenard and David A McCormick. Simulation of the currents involved in rhythmic oscillations in thalamic relay neurons. *Journal of neurophysiology*, 68(4):1373–1383, 1992.

[7] I Reuveni, A Friedman, Y Amitai, and Michael J Gutnick. Stepwise repolarization from ca2+ plateaus in neocortical pyramidal cells: evidence for nonhomogeneous distribution of hva ca2+ channels in dendrites. *Journal of Neuroscience*, 13(11):4609–4621, 1993.

[8] Walter M Yamada, Christof Koch, and Paul R Adams. Multiple channels and calcium dynamics. *Methods in neuronal modeling*, 4:97–133, 1989.

[9] James P Keener. Invariant manifold reductions for markovian ion channel dynamics. *Journal of Mathematical Biology*, 58(3):447–457, 2009.
