# Supplementary material for: Awake alpha bursting emerges as the dynamic working state in a lateral geniculate thalamocortical cell model
Source: PLoS Comput Biol. 2026 Oct 8;22(10):e1014654. doi: 10.1371/journal.pcbi.1014654 (PMC13649143; doi:10.1371/journal.pcbi.1014654)
Supplement: Supplementary file 2 [file pcbi.1014654.s021.pdf]

Editor Comments

The manuscript repeatedly associates slow (~1 Hz) oscillations and state transitions with cholinergic modulation. However, the experimental literature cited (e.g., Hughes et al., Crunelli et al.) does not support a direct causal link between cholinergic agonism and the slow oscillatory regime, as these dynamics were observed in the absence of cholinergic compounds and involved mGluR agonists. While in cortical in vitro preparations reinstating the cholinergic tone revealed a mostly synaptically generated slow oscillation (Lorincz…Crunelli, 2016 JN), in thalamic slices the cholinergic tone led to alpha bursts (Lorincz et al 2008, 2009) but not to slow oscillation even if the cells were hyperpolarized by – d.c. Please revise the text to clearly distinguish experimentally established findings from modeling assumptions or hypotheses. Also correct or qualify claims that directly attribute slow oscillations to cholinergic modulation. Explicitly state when cholinergic effects are hypothetical or indirect within the model framework.
Several reviewers raise concerns that the frequencies of slow oscillations and low-threshold bursting in the model appear higher than those typically reported in LGN slice recordings. Because the manuscript's central claim is that the model reproduces the full range of experimentally observed firing regimes, this issue must be addressed directly.
Please provide quantitative measurements (e.g., frequencies, burst rates, inter-burst intervals) for each firing regime, compare these values explicitly to known experimental ranges. Also either reconcile apparent discrepancies (e.g., species or preparation differences) or adjust model parameters and/or claims accordingly.
While the manuscript frequently states that the model reproduces experimental observations, this correspondence is not always demonstrated clearly. Please include representative experimental traces or schematic depictions (where feasible) alongside model outputs for key firing regimes or provide clearer quantitative comparisons to experimental results (e.g., ranges of frequencies, transmission probabilities, delay times).
To strengthen confidence in the generality of the findings please provide a more systematic analysis of the contribution of the model's novel components (particularly IM and ICaL), such as ablation or sensitivity analyses. Address parameter robustness and clarify whether key behaviors require fine tuning. Improve Figure clarity and presentation, reducing reliance on unprocessed example traces and adding clearer labels, units, and legends where needed. Consider including a schematic of the model summarizing ionic conductances and their interactions.

We thank both the editor and the reviewers for their in-depth reading of the manuscript and their insightful ideas, comments and questions. These comments have been extremely helpful to us in correcting manuscript claims, identifying useful literature, increasing manuscript detail, and providing interesting model extensions. We are particularly thankful for the push to further

investigate the model's "novel components" which led us to conduct new simulations that resulted in many of the new ideas presented in this revised version of the paper. Here we summarize the paper corrections/edits that have been made to our paper as well as the new content with references to the relevant editor comments.

We have corrected our claims in results sections 2.1 and 2.2 in the paper about cholinergic and glutamatergic modulation. Specifically we no longer incorrectly attribute the model's ability to produce slow oscillations to just cholinergic modulation. We provide citations that show that glutamatergic signals from the cortex can be responsible for the manipulation/modification of the background leak potassium conductance gkl in the manner described by our model. Finally, we emphasize how both of these neuromodulatory signals are insufficient for the model to reach SWS alone without additional changes in excitation signals. Finally, we have clarified within the text when claims are a model result or matching an experimental result, including making sure to cite experimental papers where appropriate. These changes are outlined in more detail in response to reviewer 3's first major comment about "Slow 1 Hz oscillations and cholinergic agonism."

We attempted to include experimental results/Figures in our paper, but found the majority of these papers to be behind paywalls and would prefer to avoid this prohibitive cost unless the editor and reviewers believe it to be absolutely necessary. We have instead, where appropriate, included the exact reference and the corresponding experimental Figure or table each time we are comparing with experimental literature. We have also included the experimental quantitative metrics when possible.

We have modified the Figures and model parameters to more correctly align with experimental results on the low-threshold bursting and high-threshold bursting properties. This is now reflected in the newly added supplemental Figures 1 and 2, which show the frequencies of bursting across parameter space. These updates and Figures are discussed in detail in response to reviewer 1's comments on Figure 1 and the delta rhythm and reviewer 3's comments on the Slow 1 Hz oscillations and cholinergic agonism.

We have now included a sensitivity analysis which investigates the high-threshold bursting properties' dependence on parameters. A sensitivity analysis is also used to strengthen our claims about how IM and ICaL are vital for model results. The sensitivity analysis shows the awake state properties of the model are robust to changes in ICaT and IH but are sensitive (and require tuning) to changes in IM and ICaL. We discuss the functional advantages of model property sensitivity to IM and ICaL in section 2.6 combined with the new analysis shown in the new Figure 12, and new supplemental Figure 16. This is discussed in detail in response to reviewer 1's comments on novel model components and reviewer 2's comments on focusing on state III versus state IV.

We have included a short new results section about the functional implications of sensitivity to modifications of ICaL, by relating our modeling work to the schizophrenia literature. This includes the new Figure 11. This section is first discussed in response to reviewer 1's comments on novel model components and then rereferenced later.

We have also throughout the results added multiple summary plots to increase confidence in our modeling results and decrease reliance on single simulation membrane potential traces. These new summary Figures are new supplement Figures 1 and 2, and newly made Figure 10. These are discussed in response to reviewer 1's comments on Figure 1 and their comments on model novel components.

We have redone Figures that have been flagged for being unclear or having poor labeling by reviewers, this includes adjustments to Figures 1,2,3,5,7, and 8. These changes are all first discussed in response to reviewer 1's comment on the respective Figure. One general change made across multiple Figures in the manuscript and supplement is the conversion of rhythmic/retinal input markers from circles to tick marks to align with common convention.

Finally we have included a new schematic in the supplement of the neuron model and the main contributions of each ionic current type. This is supplemental Figure 17, and is first discussed in response to reviewer 1's comment on Figure 1.

reviewer 1

Figure 1

Fig. 1I: The rhythm shown here appears to be around 5 Hz. Slow rhythms in thalamocortical (TC) neurons are typically < 1 Hz. Could the authors clarify how this frequency relates to experimentally observed slow rhythms?

We apologize for not picking more representative examples of sleep dynamics observed in sleep states. We demonstrate that the model is capable of producing the slow rhythms observed experimentally in thalamocortical neurons in our new Supplemental Figures 1 and 4. We have also updated the Figure 1I panel to show a <1Hz rhythm. We provide the updated Figure panels and supplemental Figures and associated captions below:

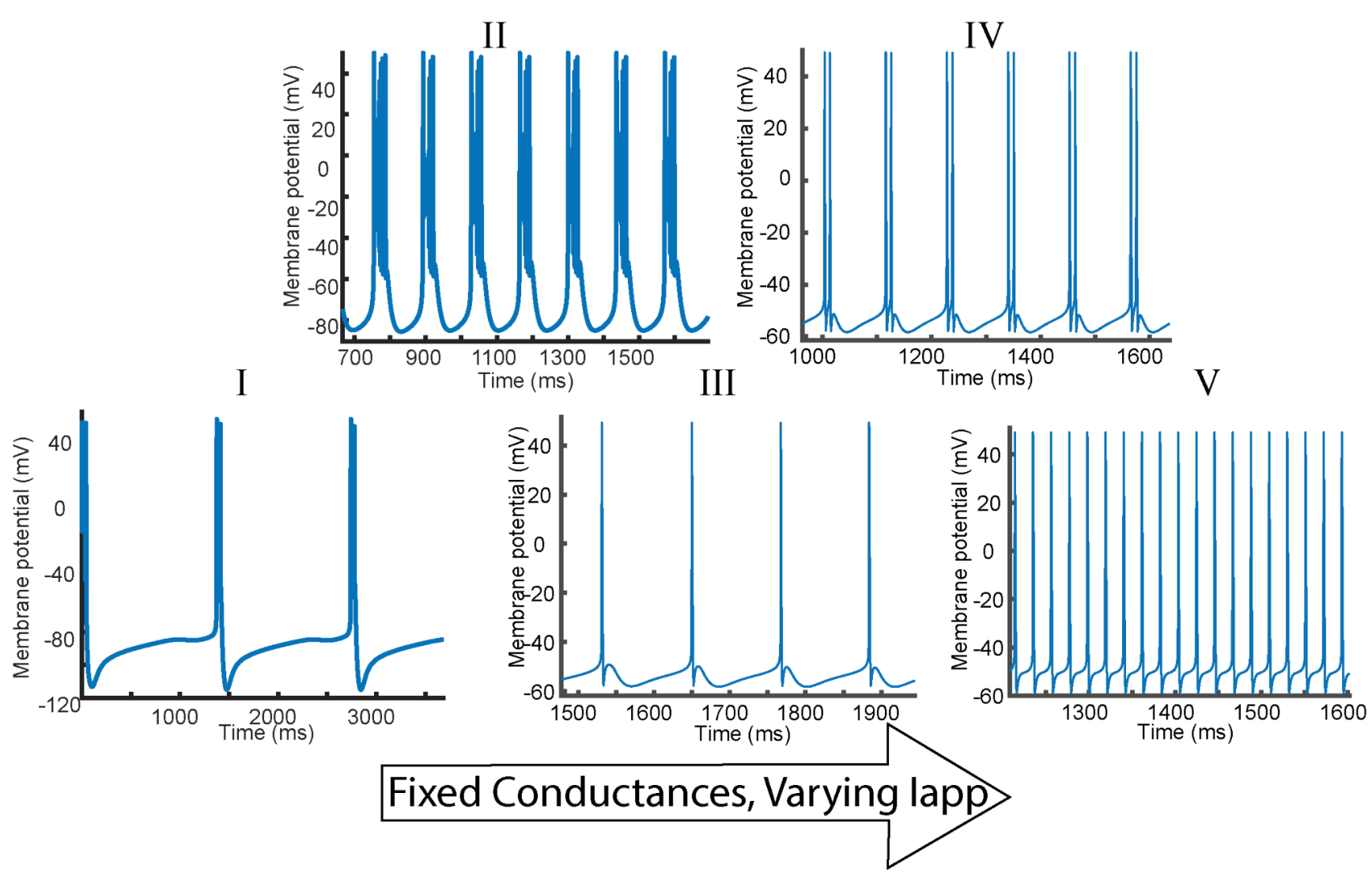

Fig 1. Thalamocortical cell model produces five distinct complex firing dynamics. With fixed ionic conductance values and increasing applied current (Iapp) from left to right, the model produces some notable firing patterns highlighted in the experimental work of Crunelli, Lorincz, and Hughes [27, 28]. In order the model dynamics are: I and II low-threshold bursting, III slow tonic firing, IV high-threshold bursting and V fast tonic spiking. A more detailed depiction (for 12 fixed values of Iapp) of the possible neuron dynamics is presented in (Supplemental Figure 1). Note, the distinction between low-threshold bursting dynamic states I and II are laid out in the next subsection. For comparison with experimental literature see: Figure 1 in [28] and Figures 1 and 2 in [37].

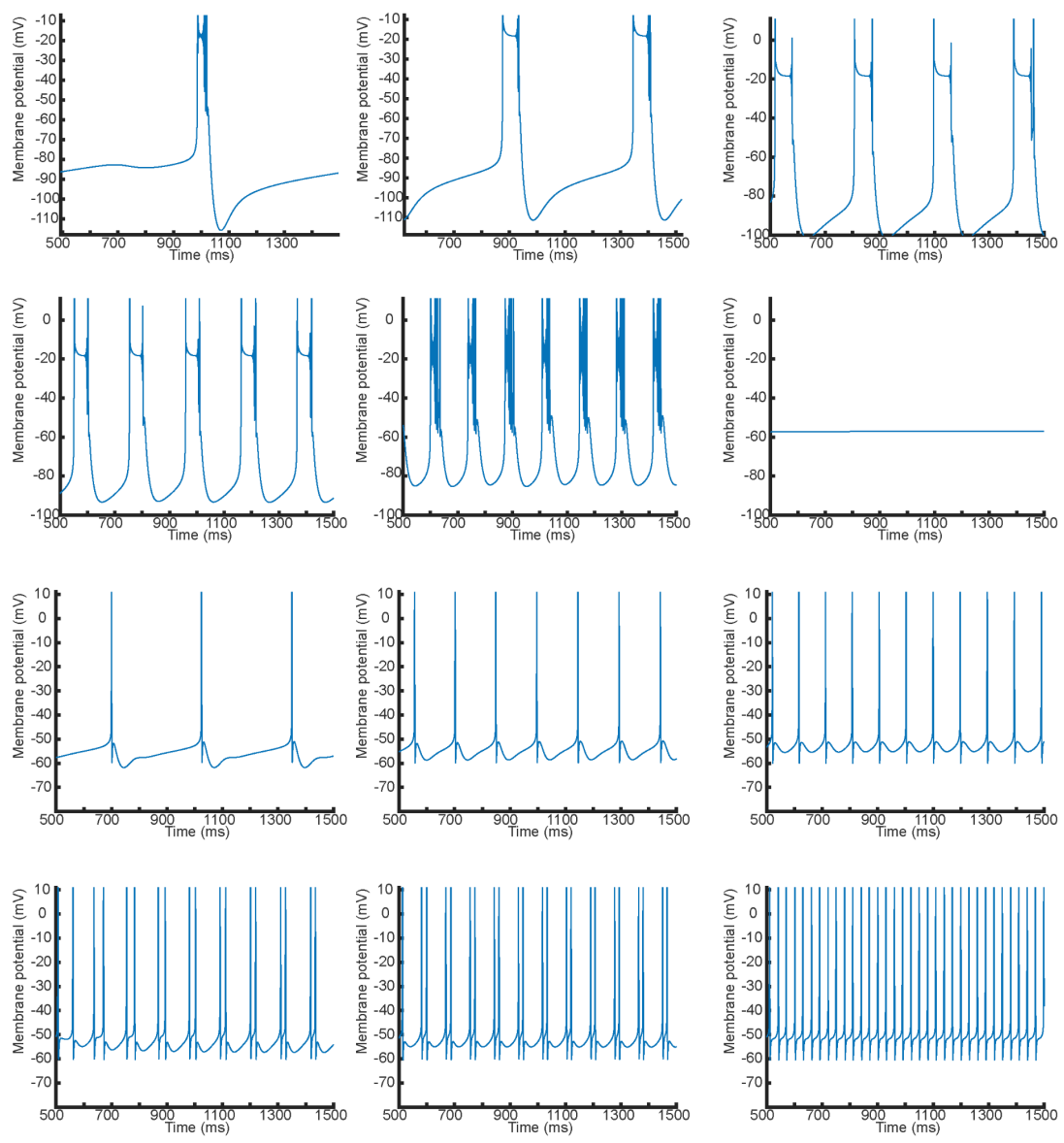

Supplemental Figure 1: TC cell model smoothly transitions between dynamic states. Moving from left to right and top to bottom, the TC model (with fixed ionic conductances, gkl = .2) response to different 12 increasing values of Iapp (-2.2, -1.8, -1, -.4, .2, .6, 1, 1.5, 2.0,.2.5, 2.9, 3.5) are presented. The low-threshold bursting frequency continues to increase until there is a transition to the quiescent depolarized state. Then there is a second transition where the single spiking begins and increases in firing frequency until the firing mode transitions to high-threshold bursting. The frequency of high-threshold bursting then continues to increase until there is a transition to fast tonic firing. Note that there are 5 distinct firing types (that can occur at multiple frequencies) presented here: low-threshold bursting, depolarized quiescence, slow tonic firing, high-threshold bursting, fast tonic firing.

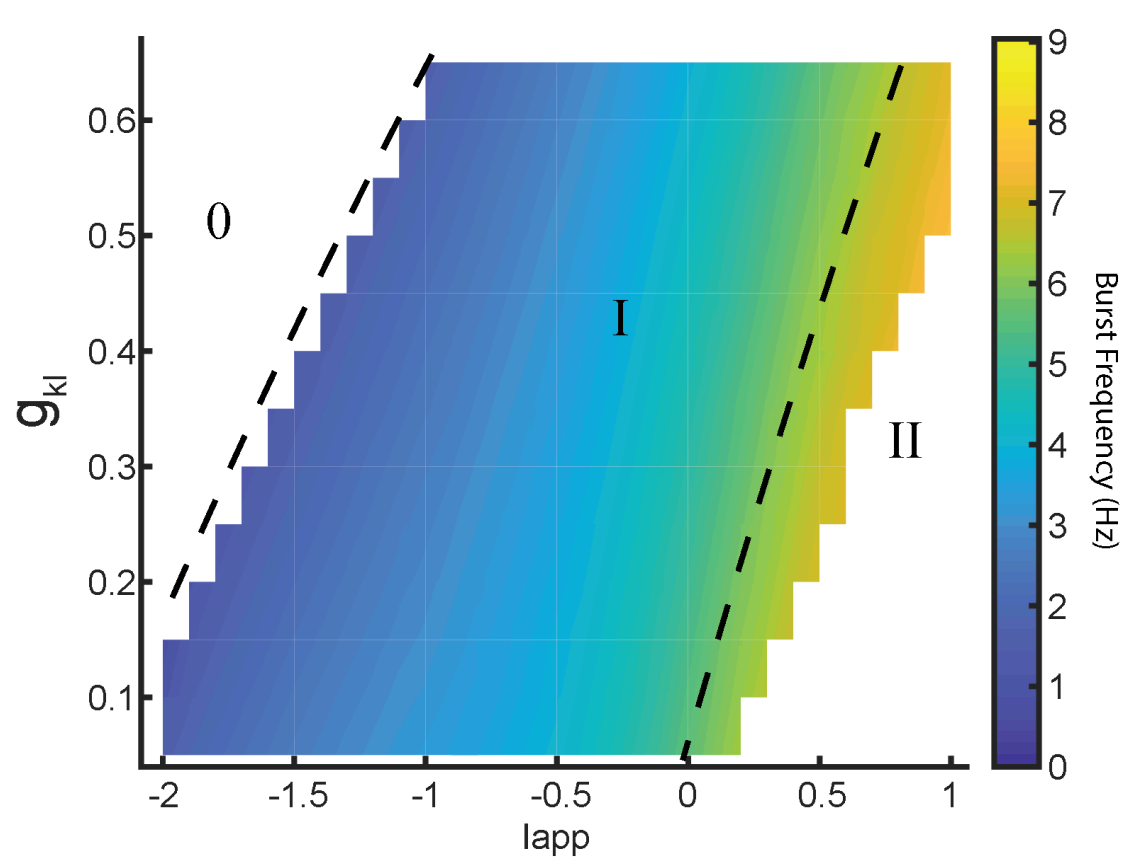

Supplemental Figure 4: Low-threshold bursting frequency increases continuously, moving from state I to state II. Burst frequency (colorbar value) is recorded as a function of changes in Iapp and scaled gkl. Slower than 1Hz frequencies are obtained at the far left end of region I, which is associated with slow-wave-sleep. The vast majority of region I produces bursting at frequencies between 1-4Hz, which corresponds to the LGN delta rhythm. Finally near and across the I/II border the LGN starts to produce higher than 4Hz bursting frequencies. These higher frequency bursts could correspond to light sleep or the type of bursting that is engaged during spindling. As discussed in section 2.2, region 2 is bistable and this frequency of bursting may not be observed without external input or specific engagement of the T-type calcium current, which may happen in vivo in response to inhibition. Note that the regions of parameter space and curves labeled here are reproductions of the regions/curves from Figure 2 in the main text.

The new manuscript text found in Section 2.1 reads:

Figures 1I and 1II show two low-threshold bursting states. The range of frequencies of low-threshold bursting includes < 1Hz frequencies that correspond to the slow wave sleep rhythms and between $1 - 4$Hz frequencies that aligns with observed delta (1-4Hz) sleep rhythms (Supplemental Figure 1) [38]. The model also suggests that low-threshold bursting at greater than delta frequencies is possible (Supplemental Figure 1), which is discussed in greater detail in the next subsection in the context of the experimental literature

Fig. 1II: The typical frequency of fast low-threshold bursting is often reported to be around 1 Hz, with a maximum of approximately 4 Hz in cat LGN slices. In contrast, the bursting activity here seems closer to 10 Hz. Unless I have overlooked something, these frequencies seem higher than those commonly observed in LGN slice recordings.

The bursting frequencies found in regions II (up to 10Hz) are higher than those commonly observed in LGN slice recordings. However, these high frequencies occur in the bistable region where the model would actually produce a depolarized quiescent state in the absence of noise or input. We explain in section 2.2 why such high frequencies would not be expected to be observed in in-vitro voltage clamp experiments by stating:

Importantly, one of the bistable states in region II is a depolarized quiescent state that is commonly observed during slice experiments when transitioning from low-threshold bursting to tonic spiking with increasing Iapp [28]. This bistability (and existence of the depolarized quiescent state) of the model is critical for explaining why frequencies higher than delta (1-4Hz) can be produced by our model even though they are not typically seen in-vitro. The model shows that experimentally observed low-threshold bursting frequencies (between 0-6Hz) occur in region I as the only stable state, while the higher (not typically experimentally observed) frequencies occur only in the bistable region II (Supplemental Figure 4). However, in the absence of external inputs that can activate the T-type calcium channel, the model suggests that

low-threshold bursts at frequencies greater than delta will be rare events: instead the silent depolarized state will emerge as is commonly seen with increasing Iapp in experiments [28].

Even allowing for potential interspecies differences (rodent vs. cat vs. human), this discrepancy remains somewhat difficult to reconcile. Could the authors explain how an LTS can be generated at ~10 Hz in Fig. 1II? It may also be helpful to adjust the model so that its bursting dynamics are constrained within more physiological frequency ranges.

We have included our new Supplemental Figure 4 (provided above) to show the full range of low-threshold bursting observable in our model. This Figure shows the dominant frequencies produced by the model in State 1 (where low threshold bursting is the sole stable state) are in the delta or sub 1Hz rhythmic band.

Finally, I suggest including a schematic of the model, with the ionic conductances and their distributions, to improve clarity and accessibility for readers.

We have made a schematic which is now Supplemental Figure 17. Within the Figure we have labeled each current according to what neuronal property they contribute to. We write in section 5.2 of the methods:

A summary diagram of our TC cell model and the primary purpose for each of the chosen ionic currents is provided in the supplement.

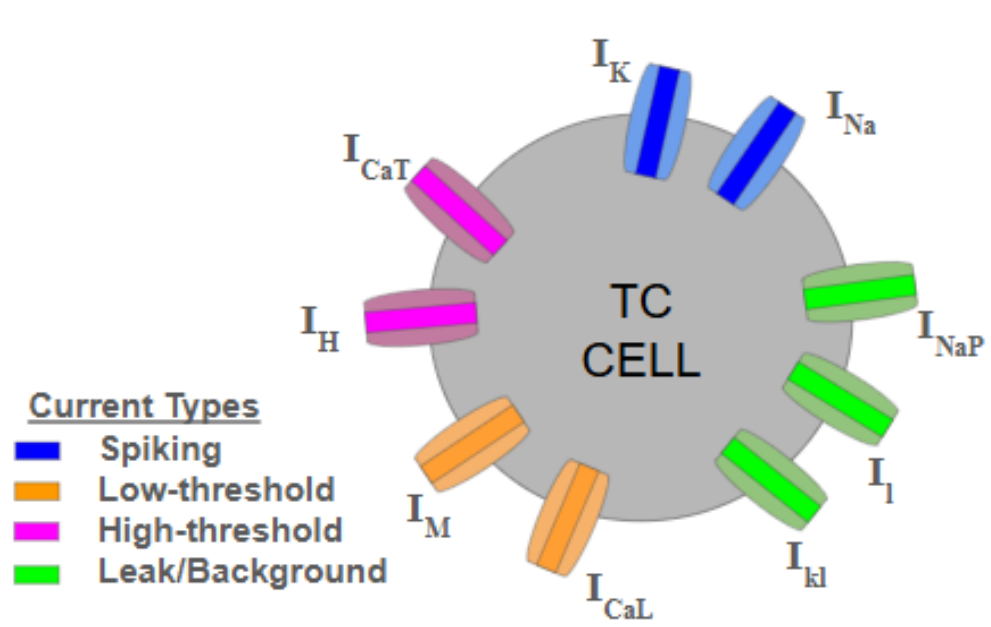

Supplemental Figure 17: Schematic depicting the TC cell model structure and the contributions of the included ionic currents. This model is a single cell, single compartment neuron model that has 9 ionic current types. These ionic currents are separated into 4 broad categories based on their primary functional role within the model: Spiking (INa and IK), Low-threshold bursting (ICaT and IH), High-threshold bursting (IM and ICaL) and Leak/background (Ikl, Il and INaP).

Note this is a single cell, single compartment model, so there is no distribution of the ionic conductances among cells or compartments.

Figure 2

This Figure describes the dynamic states of the TC neuron model in the parameter space of Iapp and gKl. The results are consistent with Figure 1 (and therefore may reflect the same

issue regarding frequency mismatch between the model output and experimental recordings).

We have altered the Figure panels in Figure II to reflect the frequency changes already discussed above in response to Figure 1 reviews. The bursting depicted in states I and II match the new panels shown above in Figure 1I and 1II.

In addition, the presentation could be improved. For example, showing transitions along a single row (or a defined trajectory in parameter space), and adding more intermediate examples, would help the reader better appreciate the continuity of the transitions between states.

We agree that more examples of the state dynamics along a single trajectory (in this case traveling horizontally through parameter space) would help show the evolution of the state dynamics. To address this, new supplemental Figure 1 shows twelve sample membrane potential plots with fixed gkl, and varying levels of Iapp. This provides more intermediate examples and should help the reader better appreciate the continuity of state transitions. This Figure and its caption are provided above and are referenced in section 2.1.

Figure 3

This Figure seems to present largely unprocessed simulation outputs. Some additional formatting and editing would greatly improve readability and interpretation.

We agree with the reviewer and thus have reformatted Figure 3 to improve readability and interpretation. We have adjusted the axis scales to be more consistent across all plots, placed the retinal spike timing input markers above the plots as is conventionally done, and taken the text out of the Figure diagram.

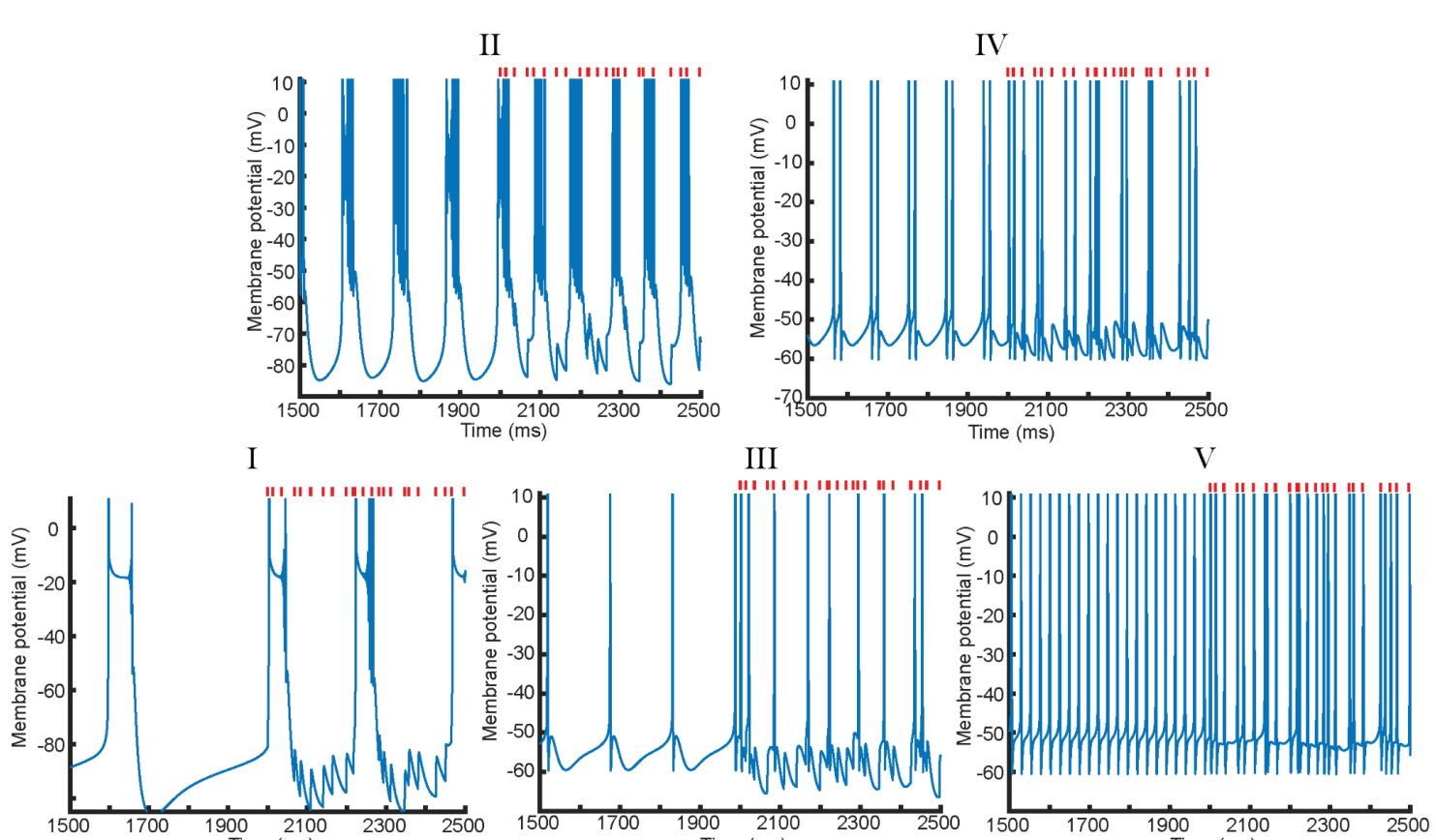

Fig 3. LGN response to retinal input changes with arousal state. The five arousal states match the order and meaning from Figure 1. Each plot shows how LGN spiking (in the 5 different arousal states) responds to an identical retinal input that is turned on at time= 2000ms. Retinal spikes occur at time points denoted by red tick marks. Note, retinal ISIs are drawn from a gamma distribution with a mean of 60 spikes/sec.

In addition, the slow and delta frequencies remain higher than expected. It would be preferable to avoid notes on how to interpret the x-axis and instead present the data in a way that is immediately clear to the reader.

We have adjusted the bursting frequency output of the plots and discussed why the model produces faster frequencies than expected (see explanation associated with Figure 1). We also removed notes on how to interpret the x-axis as the plots are now more clear.

More generally, throughout the manuscript the authors frequently refer to specific experimental characteristics (e.g., p.11, line 199; and Figures 1–3 at least). Since not all readers will be familiar with the experimental literature (notably work from the Crunelli group), it would be very helpful to include schematic experimental traces or representative recordings side-by-side with the corresponding model outputs. This would strengthen the link between the model and its biological grounding.

We agree with the reviewer that this would improve interpretability, however when we looked into including experimental results side-by-side with our work we found the experimental Figures are contained behind a paywall that is cost prohibitive to the authors. However to address this we have also included specific references to the experimental literature and note the exact Figures/tables from the experimental papers that we are referencing within the Figure captions.

Figure 4

Fig. 4D may not be necessary. Its contour could potentially be drawn directly on Fig. 4C, which would reduce redundancy and simplify the presentation.

We respectfully disagree with the reviewer on this point; we believe that drawing these additional suggested contour lines would make the Figure too messy to be usable/interpretable. We believe this presentation is the clearest way for showing that filtering overlaps with regions III and IV. We have drawn additional contour lines and provided state labels for clarity. We have also labeled the colorbars within the plot to further Figure interpretation.

Figure 5

Fig. 5D: The colored ISIs are difficult to see and are not clearly defined in the legend. In addition, it is unclear whether these results are quantified. Providing clearer definitions and/or quantitative measures would help improve interpretability.

We agree that the current labeling is unclear and so we have adjusted Figure 5 and the labeling to provide clarity. The color labeling in the ISIs has been removed to eliminate possible confusion. The labels in Panel B are used to indicate an example of the three different ISI

categories while the labels in panel C are used to give the full range of ISIs for each category ISI_S=0-10ms, ISI_M=10-30ms and ISI_L=>30ms. The new Figure and caption is given below:

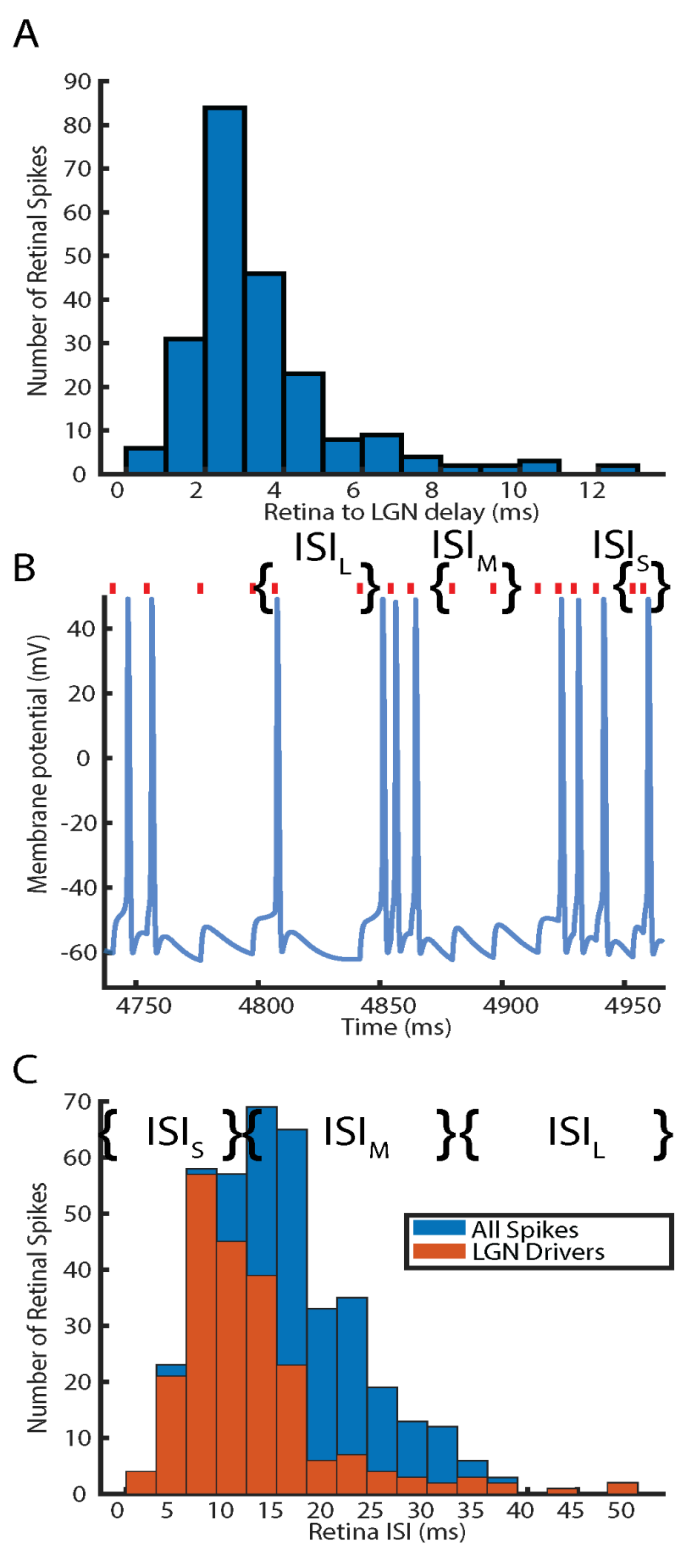

Fig 5: LGN spike generation in the awake state IV is heavily influenced by retinal ISI. A) Distribution of delay times between each LGN spike and the preceding retinal spike. B) Membrane potential of the LGN TC cell plotted against time. Red tick marks denote time stamps of the incoming retinal spikes. Curly braces depict example retinal ISIs from the three distinct time length categories: short (ISI_S, 0-10ms), medium (ISI_M, 10-30ms) and long (ISI_L, >30m). C) Distribution of retinal interspike intervals. The ISIs for the full retinal spike train are plotted in blue. The overlapping orange histogram depicts the ISIs between two retinal spikes where the second spike is an LGN spike driver. Curly braces here depict the three distinct time length categories.

Figure 6

Fig. 6D appears largely redundant with Fig. 6B and could potentially be omitted or integrated.

Please see our response for Figure 4 above. The same reasons we give in our response to the suggestion of potentially omitting Fig 4D are also applicable for Fig 6D.

Figure 7

It might be clearer to represent retinal input using a vertical tick mark, rather than circles, to improve readability and consistency with common conventions.

We agree with the reviewer and therefore have converted the retinal inputs to vertical tick marks to improve readability and consistency with common conventions. We have made this change across all Figures that include retinal/spiking input.

Figure 8

Fig. 8A and 8C are difficult to interpret in their current form. Fig. 8B would benefit from clarification (e.g., improved labeling and explanation). Fig. 8D resembles a scatter plot, but the meaning is not clear: additional explanation, interpretation, and the inclusion of a trend or quantitative analysis would help the reader understand what is being conveyed.

Figures 8A and 8C have been adjusted to improve interpretability. They are now both sample plots of entrainment, with 8A showing entrainment to a periodic signal, and 8C showing entrainment to a nested theta-theta gamma signal. Furthermore we have moved the brackets in 8C for increased clarity. We have changed the 1:1 entrainment line colors to orange to eliminate any possible confusion with overlapping colors for the tick marks. The corresponding text within the manuscript is now written as:

Figure 8A shows an example of the thalamus successfully transmitting a high beta (27Hz) frequency input: in fact, inputs between 7Hz and 30Hz result in perfect entrainment (overlap with orange 1:1 line in Figure 8B), albeit with different output firing types (single spikes, doublets, or mixed mode) (Supplemental Figure 9). In contrast, for all frequencies between 30Hz and 70Hz, the ratio of input to output frequency falls beneath the orange 1:1 line, indicating that gamma frequency inputs are not faithfully transmitted. We find that the thalamic output is limited to frequencies below 30Hz in response to any periodic input at frequencies between 30Hz and 70Hz (Figure 8B)

We Include Supplemental Figure 9 (which is unchanged from the original submission but is cited within the text above) for ease of understanding.

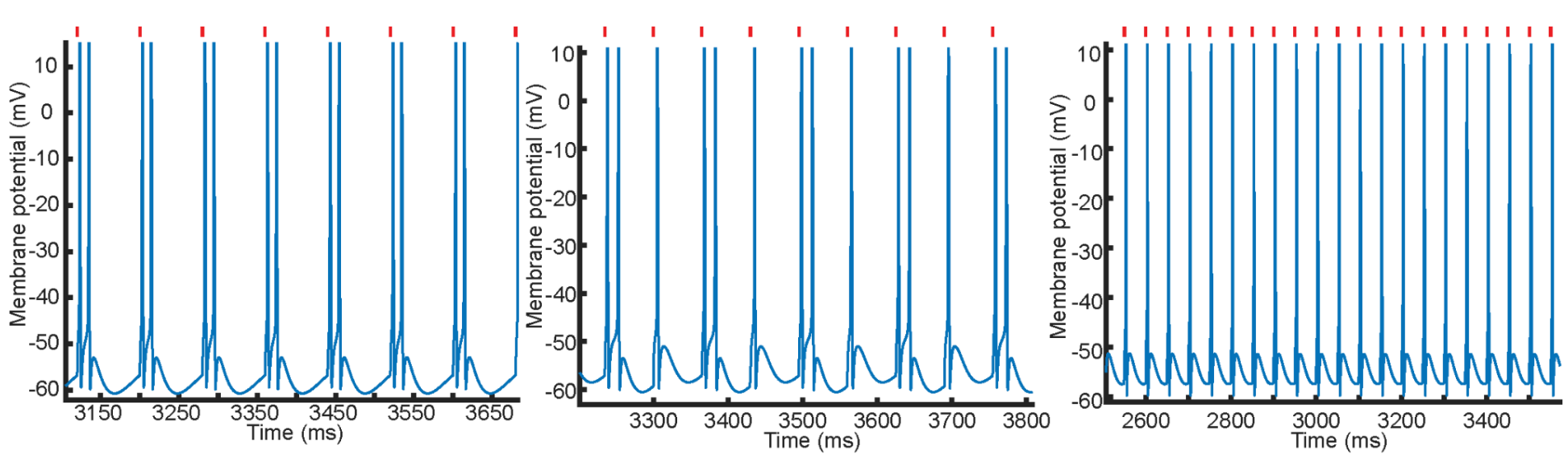

Supplemental Figure 9: Three distinct firing types that occur during thalamic 1:1 entrainment to periodic excitatory inputs. Successful transmission of periodic inputs depends on the level of entrainment. Note,

entrainment is measured by comparing the ratio of input frequency to output frequency. The output within a single cycle of a periodic output could be a burst or a single spike. This means that there may be more output spikes than input spikes, but the neuron could still be considered to be entrained. As an example, during 1:1 entrainment to excitatory periodic inputs the TC cell exhibits three output types: entrained output as doublets (left), entrained output as mixed mode, meaning alternating doublets and singlets (middle), entrained output as single spikes (right). The mixed mode entrainment is interesting as it represents a firing model shift of the TC cell and can result in minor deviations from perfect 1:1 entrainment. The incoming input frequencies are 14Hz (left), 16Hz (middle), and 20Hz (right). Red dots denote timing of excitatory input spikes

Finally we have altered the axis labels of 8D to help with interpretability. The new axis label reads: "Entrainment to Gamma Frequency Inputs " Additionally we have adjusted the Figure caption and text to aid the reader with understanding. The new Figure and caption are given below:

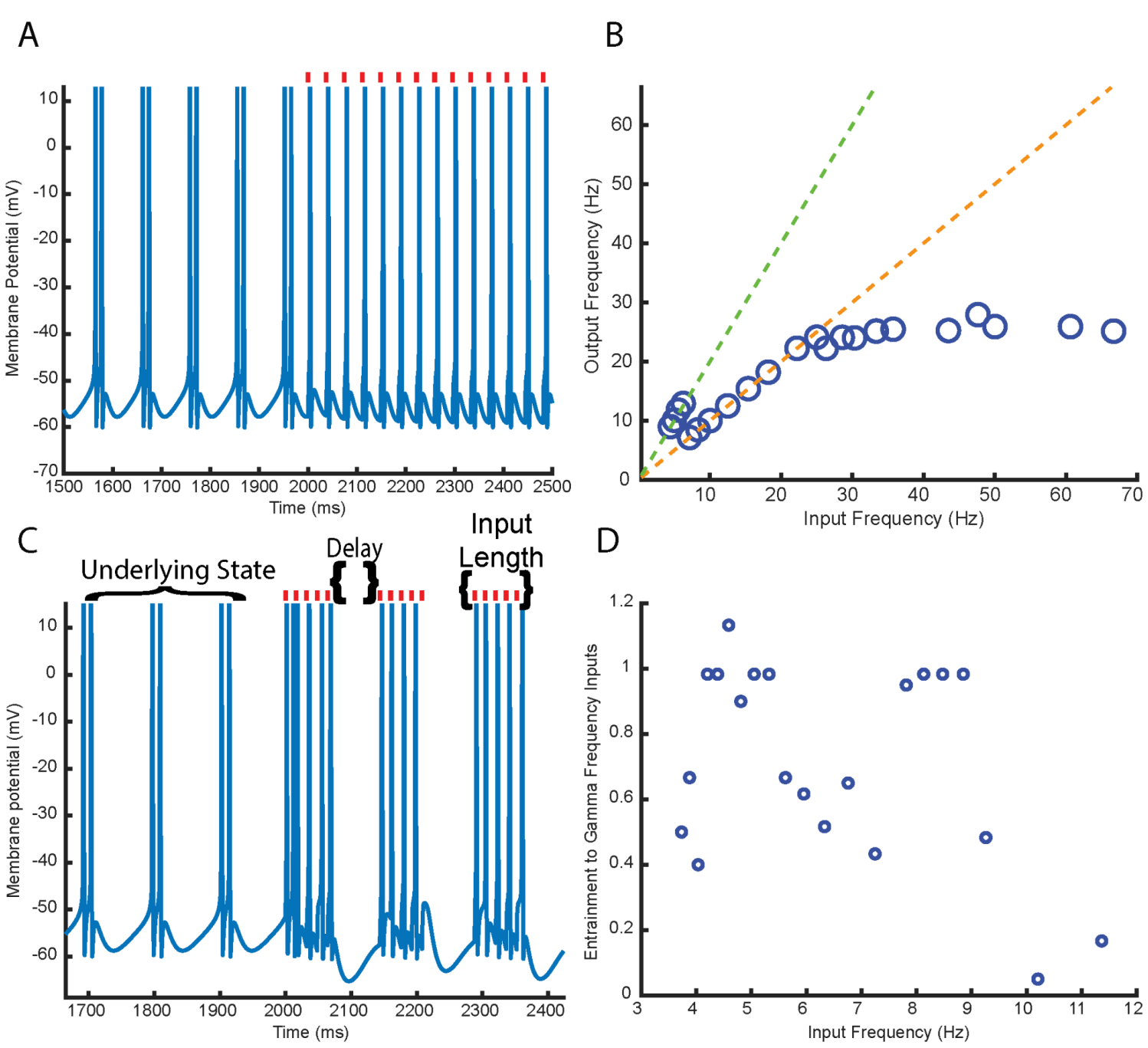

Fig 8. Awake thalamus successfully entrains to periodic excitatory alpha and beta rhythms but passes theta and gamma only as a nested rhythm. A) Sample trace of the TC cell (in state IV) response to a periodic 27Hz spiking frequency input; the thalamus is perfectly entrained to the signal. Red tick marks denote timestamps of incoming excitatory spiking inputs. B) Summary plot showing the number of thalamic burst (or single spike) outputs per second in response to periodic rhythmic excitatory input. Line colors correspond to 2:1 partial entrainment (green) and 1:1 entrainment (orange). C) Sample trace of neuron's response to a nested theta-gamma (8/66.5Hz) input showing near perfect entrainment to incoming gamma spikes. Entrainment depends on three features that are labeled here: the silent period between the incoming bursts of spikes (labeled as Delay), the combined frequency and number of the incoming fast inputs (labeled as Input Length) and the underlying state of the neuron (labeled as Underlying State). The impact of each of these features is analyzed/discussed within the text. Red tick marks denote timestamps of incoming excitatory spiking inputs. D) TC cell entrainment to the spiking gamma frequency inputs as a function of the incoming slow rhythm frequency. The dependent variable is calculated as the number of TC spikes that match the incoming gamma frequency divided by the number of incoming spikes at the gamma

frequency. A value of 1 here indicates 1:1 entrainment to the gamma frequency inputs. Gamma frequency inputs are fixed at 66.5Hz, while the slow timescale (denoted by Delay in panel C) is varied to generate a slow rhythm frequency between 3Hz and 12Hz.

The accompanying description in the text in section 2.7 reads:

Excitatory gamma transmission is most successful when embedded within theta frequency oscillations and shows decreased effectiveness if embedded within alpha (> 9) or delta (< 4) rhythms (Figure 8D). Interestingly, within the theta frequency band, there are two distinct peaks for perfect 1:1 gamma entrainment found at 4-5.5Hz and 7.5-9Hz (Figure 8D, Supplemental Figure 10). This is noteworthy, as distinctions between high and low theta have been made previously in relation to working memory, behavioral tasks and spatial cognitive processes [65–67].

Figure 9 Whenever the authors refer to "bursts" in this Figure (and elsewhere), it would be helpful to specify whether these are high-threshold bursts (low frequency) or low-threshold bursts (high frequency), as this distinction is not readily apparent from the Figure.

We have updated throughout the text to better clarify which type of burst we are referring to when possible. In some cases, such as the outputs exhibited in Figure 9, the bursts are being driven by external inputs. This means the properties of the bursts (spike frequency and minimum membrane potential) can fall somewhere between the experimentally defined windows for the two different types of bursts discussed here (low-threshold versus high-threshold bursts). This makes it difficult, especially in the case of bursting in response to rhythmic input, to define which type of burst is being produced in our model. In cases where the type of bursting is unclear, we refer to these bursts as being input driven. We also find an intermediate burst type in our analysis involving schizophrenia when we increase the L-type calcium current. Here we write:

The first observation from Figure 11A is that the increase in L-type calcium conductance changes the baseline high-threshold alpha frequency bursting of state IV into a bursting output occurring at theta frequency. While the theta bursting output has a faster ISI and more spikes per burst in comparison with the high-threshold alpha, it also does not fully resemble low-threshold bursting, implying an intermediate bursting type.

Major revisions

The key contribution of the manuscript is the proposal of a new thalamocortical neuron model. However, at this stage, the manuscript would benefit from additional work in order to strengthen confidence in the model and improve its interpretability.

In particular:

1. The model does not appear to reproduce the delta rhythm (~1–4 Hz) as reported extensively in previous experimental and modeling studies. This point should be addressed directly.

We thank the reviewer for noting this and have now shown that our model does reproduce the known experimentally observed rhythms. We have addressed this point directly by decreasing Iapp in the model to show that the model reproduces both the slow (<1 Hz) rhythm and the delta rhythm. This is shown by our new Supplemental Figures 1 and 4 which are provided above in our response to the reviewer's comments on Figures 1 and 2. Figures 1 and 2 have also been revised to show the slow and delta rhythms (see above). In the main text in section 2.1 we write:

"Figures 1I and 1II show two low-threshold bursting states. The range of frequencies of low-threshold bursting includes <1 Hz frequencies that correspond to the slow wave sleep rhythms and between 1-4 Hz frequencies that aligns with observed delta (1-4 Hz) sleep rhythms" The model also suggests that low-threshold bursting at greater than delta frequencies is possible (Supplemental Figure 1), which is discussed in greater detail in the next subsection in the context of the experimental literature.

2. The manuscript does not sufficiently explore the impact of the model's "novel" components. Systematic analysis of how these components influence firing regimes and rhythmicity would significantly strengthen the work.

We have now conducted a more systematic analysis in the paper showing how the model's novel components impact the model results. This analysis includes the entire new section 2.9, which describes how the novel regime (high-threshold bursting) in the model is influenced by the key non-spiking currents and specifically shows how the L and M currents alter the properties of the high-threshold bursting within the model. We write:

Figure 12 shows that high-threshold bursting is robust to changes in gH and gCaT , but sensitive to changes in gCaL and gM . Variations in gH or gCaT by 10% resulted in less than a 5% change for all three of the high-threshold burst properties. However, varying gM by 10% resulted in a near equivalent percent change (with opposite sign) in the high-threshold bursting frequency. Notably, we find the model was most sensitive to changes in gCaL, a 5% change in gCaL consistently resulted in a drastic shift in firing properties, or eliminated high-threshold burst firing altogether (Figure 12 and Supplemental Table 1). These results are striking as IM is modulated during attention and arousal while ICaL is implicated in the development of neuropsychiatric diseases (see previous subsection). The extreme sensitivity of model properties to changes in gM and gCaL suggests that the thalamus tightly regulates these currents to allow for different properties depending on functional context. For example, high sensitivity of the model to gM and gCaL may permit alterations of the filtering window or entrainment ranges established by these two currents to flexibly enable the passage of signals which are normally not transmitted.

We provide Figure 12 and its caption below:

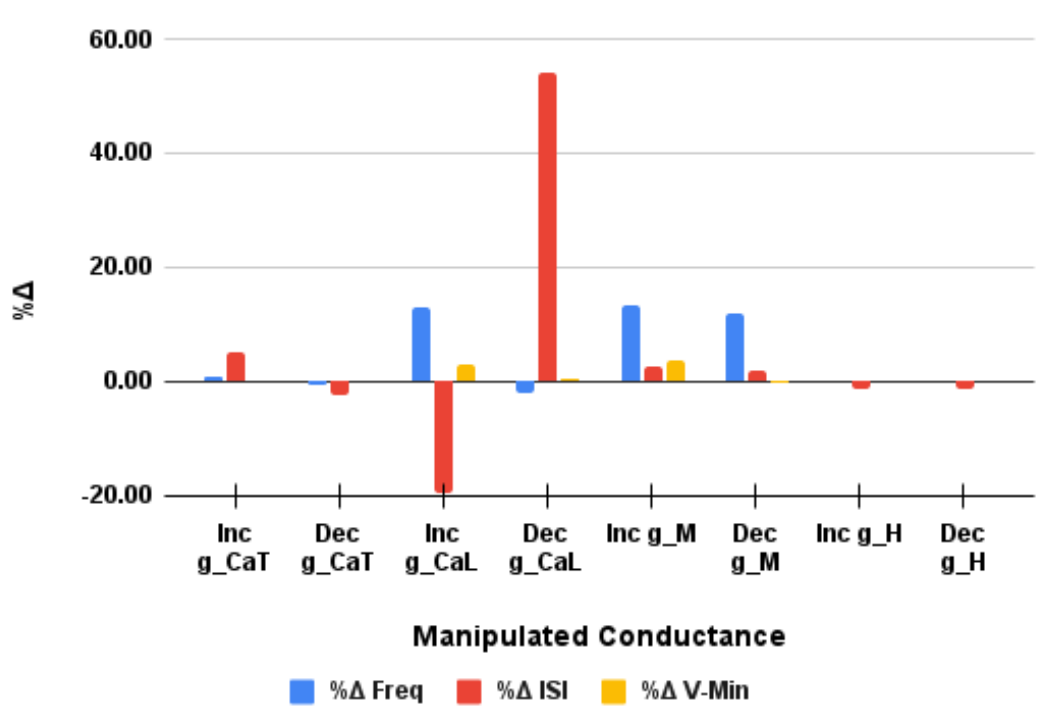

Fig 12. High-threshold bursting exhibits high sensitivity to changes in gCal and gM but not gCaT or gH . The average (across four parameter sets) percent change (%Δ) from the recorded value for a control parameter set of each of the three key high-threshold bursting properties (frequency:blue, ISI:red, and minimum voltage:yellow) is plotted as a function of manipulated ionic conductance. Conductances were varied in both directions (labeled here as Inc for increased or Dec for decreased). The full non-averaged data set containing the values for each of the four chosen parameter sets is presented in Supplemental Table 1.

We have described in the manuscript how the novel model components (the M and L currents) dictate the filtering properties of the neuron in section 2.6 highlighted by when we write:

"Figure 7 reveals that the inactivation time constant of ICaL and the decay time of IM are key factors for determining the transmission probability in the awake state for short and medium length retinal ISIs respectively."

Additionally, we have the new disease focused section 2.8 that describes what happens to the model and its rhythmic entrainment properties with changes to the L current. This is shown with our new Figure 11:

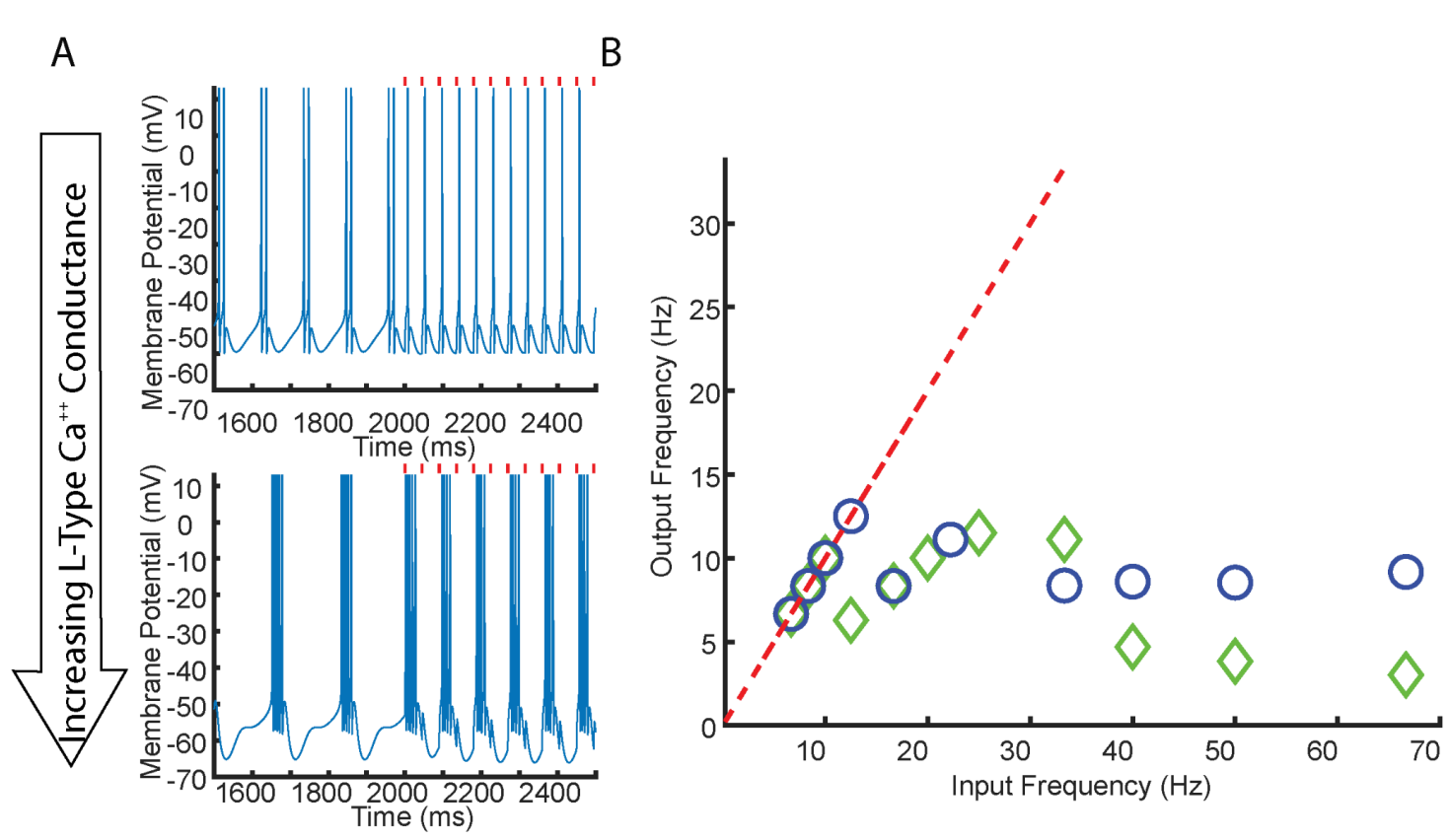

Fig 11. Increases in L-type calcium conductance change the baseline firing type and entrainment properties of the state IV thalamus. A) Membrane potential plots showing the underlying state dynamics and response to periodic excitatory inputs (at 24Hz) for an unaltered thalamocortical neuron (top) and a thalamocortical neuron with

increased gCaL (bottom). External spiking input is turned on after 2000ms, with the timing of the inputs denoted by the red tick marks. B) Summary plot showing number of thalamic input-driven-burst (or single spike) outputs per second in response to incoming periodic rhythmic excitatory (blue circles) or inhibitory (green diamonds) signals. These responses are for a neuron initialized in state IV but with increased gCaL.

We describe the results of Figure 11 with the following text of section 2.8:

The model suggests that increasing gCaL alters the underlying baseline firing properties and eliminates the robust entrainment features of the state IV thalamus. The first observation from Figure 11A is that the increase in L-type calcium conductance changes the baseline high-threshold alpha frequency bursting of state IV into a bursting output occurring at theta frequency. While the theta bursting output has a faster ISI and more spikes per burst in comparison with the high-threshold alpha, it also does not fully resemble low-threshold bursting, implying an intermediate bursting type. This alteration in firing behavior also affects the response of the thalamus to periodic excitatory and inhibitory inputs. While the unaffected state IV TC cell produced 1:1 entrainment to frequencies from 7-30Hz (excitatory) and 8-16Hz (inhibitory) (Figures 8 and 9), here the model fails to entrain to any input above 12Hz (Figure 11B). Furthermore, in the gCaL altered TC neuron, the output frequencies (y-axis value) are restricted to values between 4Hz and 12Hz, regardless of input type (excitatory or inhibitory) and frequency (Figure 11B). This result suggests that this genetic mutation in the L-type calcium channel gene CACNA1 could lead to a rigidity in thalamic processing that may help explain schizophrenia deficits in cognitive flexibility and task switching [75, 76].

We also show more clearly how the rhythmic firing entrainment properties of the model are dictated by model state. To do so we include a new summary Figure 10 in section 2.7.

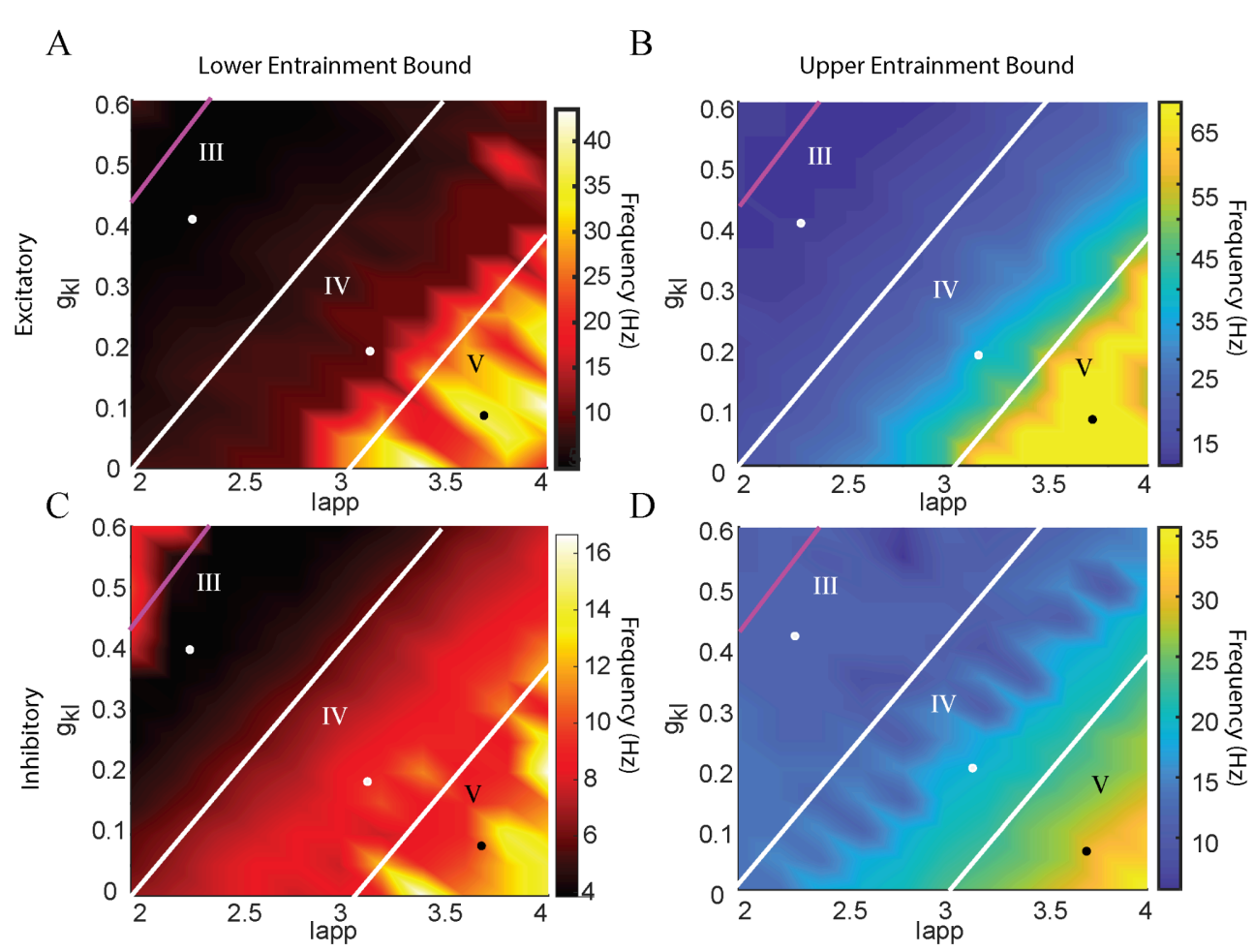

Fig 10. Possible entrainment range widens as the underlying dynamics of the model are moved from state III to state IV. A) Lower frequency limit that the model is capable of entraining to for excitatory periodic inputs. B) Upper frequency limit that the model is capable of entraining to for excitatory periodic inputs. C) Same as A, but for inhibitory periodic signals. D) Same as B, but for inhibitory periodic signals. All entrainment frequencies (given in Hz) are presented as color intensity (depicted on colorbar) for varying values of Iapp and potassium leak conductance (scaled gkl). All awake state regions (III, IV and V) and

delineating borders are redrawn for clarity. A representative point in each region is highlighted for ease of description and comparison of the entrainment properties within and between regions. Model response to periodic inputs outside of the entrainment limits in states III and IV can be found in Supplemental Figure 13.

The corresponding text describing Figure 10, and what it says about entrainment limits across state is given below:

Perturbations of the underlying dynamic state suggest that state IV is optimal, when contrasted with states III and V, for entraining to slower (alpha and beta) periodic excitatory rhythmic signals. More specifically, shifting to a faster high-threshold bursting underlying frequency by increasing excitation (moving from state III to state IV through increases of Iapp or decreases of gkl) results in a broader range of excitatory periodic input frequencies that the model can entrain to. Across the entire parameter space devoted to both regions III and IV, the lower bound for entrainment is roughly in the theta band (6-8Hz) until the IV-V border (Figure 10A). However, moving from state III into state IV, the upper bound of entrainment drastically increases from near 15Hz (region III sample point) to near, or above, 25Hz (region IV sample point) (Figure 10B). Note that once the model reaches state V, both the minimum and maximum entrainment frequencies drastically change and are constrained to be in the gamma frequency (30-70Hz), no longer entraining to alpha or beta (Figures 10A and B).

For periodic inhibitory signals across regions III and IV, we find that increasing external excitation (Iapp) increases both the upper and lower bound for possible entrainment frequency. The lower bound is increased minimally from ~ 5HZ (region III sample point) to ~ 8Hz (region IV sample point) (Figure 10C). However, the upper bound is limited to ~ 10Hz in region III but can reach ~ 20HZ in region IV (Figure 10D). This implies that an increase in baseline high-threshold bursting frequency broadens the range of possible entrainment frequencies, critically allowing for the passage of inhibitory low beta signals. Once again, note that upon transition to state V, there is a large shift in entrainment properties: the model no longer entrains to inhibitory theta or alpha frequencies, but can reproduce an incoming inhibitory gamma (Figures 10C and D).

More broadly, many Figures consist of example traces without sufficient generalization or quantification (e.g., frequency, amplitude, variability), and without parameter sensitivity analysis. Since neuronal models often rely on fine-tuning of key parameters, the manuscript would greatly benefit from including an analysis of the most influential parameters and an evaluation of the robustness of the reported behaviors.

We agree with the reviewer and thus have now included a sensitivity analysis in section 2.9 within the paper. Much of this is described above in response to exploring the novel components

of the model and describing the low-threshold bursting properties associated with Figures 1 and 2. Our response also includes the new Figures 10, 11, 12 and Supplemental Figures 1 and 4, all of which are described above.

We also have included a sensitivity analysis (section 2.9) for retinal filtering properties of the model. We write:

Sections 2.4-2.6 characterize mean retinal transmission properties and how the filtering of different retinal ISIs is influenced by gCaL and gM; here we show that these features are robust to changes in gCaT and gH , and that the conclusions of sections 2.4-2.6 hold under different levels of baseline model excitation (different parameter sets from the Iapp vs gkl parameter space are chosen). Supplemental Figure 16A shows that the transmission probability curve for different length ISIs is largely unaltered by changing either gCaT or gH . More specifically, under these parameter variations, the model still predicts a high passage of short and long ISIs with a strong filtering out of the medium length retinal spike times. Additionally, although the total transmission probability increases with increased excitation (moving from state III to state IV), the timescales of retinal ISIs preferentially passed through the thalamus are similar throughout regions III and IV of Iapp vs gkl parameter space with the greatest percentage of filtered out ISIs occurring in the 20-40ms range Supplemental Figure 16B.

This new text is associated with Supplemental Figure 16B which is provided here:

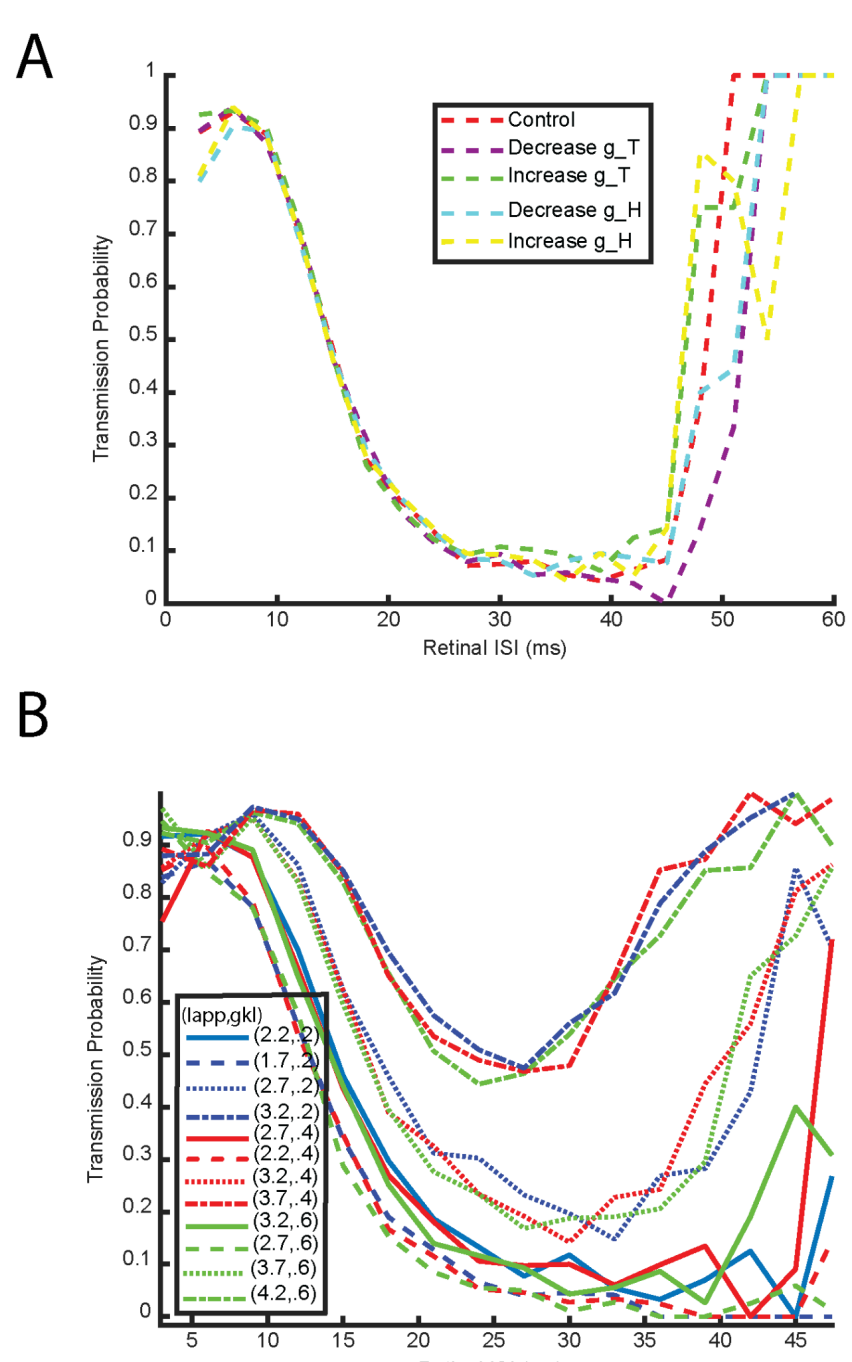

Supplemental Figure 16: Retinal filtering is robust to changes in gH and gCaT and produces a similar filtering window across regions III and IV Iapp vs gkl excitation space. A) Transmission probability as a function of Retinal ISI plotted for the control parameter set and 4 variations of this parameter set which are: 10% decrease in gCaT (purple), 10% increase in gCaT (green), 10% decrease in gH (blue), and 10% increase in gH (yellow). The four variations of the control parameter space produce a similar transmission probability curve. B) Transmission probability as a function of Retinal ISI plotted for 12 distinct parameter sets from within regions III and IV of Iapp vs gkl parameter space. Parameter sets were found by fixing a value of gkl and then taking four .5 increments of Iapp. This was repeated for 3 separate values of gkl yielding the total of 12 parameter sets. We find that the effect of changing Iapp can be counteracted by changes in gkl, and that throughout the entirety of the parameter space tested, the 20-40ms window of retinal ISIs are the most likely to be filtered out as described in the main text. The

lowest (smallest transmission probability) two groupings of curves (by line type) are from region III and the higher two groupings of curves are from region IV in parameter space.

Finally, although the manuscript includes many references to experimental literature, it remains difficult for the reader to assess how well the model reproduces the specific experimental phenomena claimed. The authors could strengthen this point by presenting representative experimental data or schematics side-by-side with model outputs, or by including explicit quantitative comparisons to the relevant literature.

Please see the response to the Figure 3 review which addresses this point.

reviewer #2: McGahan et al. present a novel LGN thalamocortical cell model. It has a number of strengths:
1. It reproduces diverse experimentally observed firing patterns, providing insights into potential underlying mechanisms of state transitions at the ion channel level.
2. It provides insights into how retinal input is processed (blocked, filtered or faithfully transmitted) by the LGN in a state-dependent manner.
3. It links thalamocortical processing to neuromodulatory functions (primarily cholinergic).
4. It establishes possible mechanisms of oscillation entrainment in the thalamus in a frequency-specific manner, including the testing of nested oscillations.

The manuscript is a thorough and important piece that brings seminal electrophysiology work from the Crunelli lab and recent genetic data together in one relatively simple model that nevertheless produces complex dynamics. I think this will be an important reference point for thalamocortical modeling and information processing in the future. I only have a few points aiming to provide further insights as well as ease the understanding of the manuscript for the reader.

We thank the reviewer for their detailed reading of our manuscript and providing insights for easing interpretability for future readers.

1. It would greatly increase the appeal of the model if the Authors could provide a direct demonstration of how the model reproduces experimentally observed dynamics, e.g. in Figure 1, even if only by replotting previous experimental results.

We responded to a similar comment from reviewer 1 earlier, and we repeat the response here:

We agree with the reviewer that this would improve interpretability. However when we looked into including experimental results side-by-side with our work we found the experimental Figures are contained behind a paywall which is somewhat cost prohibitive to the authors. Thus

we have also included specific references to the experimental literature and the experimental Figures/tables we are referencing within the Figure captions.

2. It is stated multiple times that the channel constitution is based on recent genetic data (e.g. from Allen Brain Institute). However, to make this claim clearer, more details are needed on how exactly the genetic data informed the model, preferentially even demonstrated visually.

We have clarified in the methods section 5.2 how we use genomic datasets to inform model choices.

"More specifically, we look up the gene associated with each ion channel chosen to be included in the model and verify positive expression within the LGN and the thalamus more broadly. It is important to highlight that we are not modeling/including ALL channels expressed within the thalamus, but are instead searching for a minimal subset that reproduces the dynamics of interest."

We also describe the optimization process in 5.3 by writing:

"The upper and lower bounds for the conductances gj set during the parameter search were provided by previous modeling studies of the thalamus and the cortex (in the case of the M-current where previous thalamic modeling studies are lacking)."

3. There is some ambiguity in focusing on states III and IV vs. only on state IV. For instance, Fig. 5 could show both III and IV, given that the corresponding text claims they are similar, suggested also by the title of section 2.5.

We agree with the reviewer that we could conduct a more comprehensive analysis of the model's capabilities in states III. To address this we have expanded our filtering and entrainment analyses to demonstrate how the trends and properties associated with state IV change or stay the same in state III. This includes showing that the same sets of retinal ISIs are filtered out in state III but with far fewer percentage of spikes making it through (Figure 4 and Supplemental Figure 8). With rhythmic entrainment, our results demonstrate that State IV exhibits a greater entrainment range than state III for both periodic and nested rhythmic oscillations as discussed in section 2.7:

Excitatory Periodic:

Across the entire parameter space devoted to both regions III and IV, the lower bound for entrainment is roughly in the theta band (6-8Hz) until the IV-V border (Figure 10A). However, moving from state III into state IV, the upper bound of entrainment drastically increases from

near 15Hz (region III sample point) to near, or above, 25Hz (region IV sample point) (Figure 10B).

Inhibitory Periodic:

The lower bound is increased minimally from ~ 5HZ (region III sample point) to ~ 8Hz (region IV sample point) (Figure 10C). However, the upper bound is limited to ~ 10Hz in region III but can reach ~ 20HZ in region IV (Figure 10D). This implies that an increase in baseline high-threshold bursting frequency (moving from state III to IV) broadens the range of possible entrainment frequencies, critically allowing for the passage of inhibitory low beta signals

Nested Rhythms

Furthermore, while inhibitory nested inputs appear to produce a similar output in states III and IV, only state IV successfully transmits all of the incoming excitatory gamma spikes (Supplemental Figure 14).

We have described these changes in more detail in response to comments from reviewer 1 regarding sensitivity analysis and analysis of novel model components. We highlight the new included Figures 10, 11, 12, and Supplemental Figure 16 and then refer the reviewer to the associated comments/descriptions given above as a response to reviewer 1's comments about novel components and sensitivity.

Finally, the alpha frequency is considered to be the normal baseline thalamic rhythm during the awake behaving animal based on experimental data. Thus we highlight the processing that occurs during State IV more than in State III, because state IV is where we find that the high-threshold bursting that underlies the baseline rhythm occurs at an alpha frequency. We write at the beginning of section 2.7:

This subsection focuses primarily on signal processing during the alpha generating awake state IV (as this is the experimentally observed thalamic baseline awake state/rhythm), but will discuss rhythmic signal processing in states III and V at the end.

4. The different role of the T-type Ca channels compared to previous models is a striking feature of the model that would deserve more focus. It is contemplated in the methods section 5.1, but maybe that discussion would more belong to intro/discussion sections. Is this difference rooted in the genetic data that were considered, and if yes, how?

We are unclear what the reviewer means by the new role of the T-type Ca channels in the model. Specifically in our model the low-threshold T-type Ca channel plays the same role in low-threshold bursting and sleep rhythms as in previous papers/models. We discuss in section 5.1 how for awake high-threshold bursting dynamics, previous models used a high-thresholdT-type Ca current that was phenomenologically constructed with kinetic properties not fit to data.

Furthermore it was not clear which calcium gene this high-threshold T-type current was referencing and what its expression levels were. Thus our model simply does not include a current which we believe does not have sufficient experimental evidence for its inclusion. For these reasons we do not create a big discussion point about this topic, other than acknowledging the limitations for these previous models. If the reviewer is referencing a different role of T-type channels, or the role of the L-type current, we would appreciate further clarification.

5. The separation of theta sub-bands in Fig.8D is interesting. What could underlie this in the model?

In the model the separation into theta sub bands (4–5.5Hz and 7.5-9Hz) relates to how the inputs are timed according to the ongoing alpha phase. The interaction between the underlying alpha dynamics and the two currents (IM and ICAL) prominently responsible for generating these dynamics disrupts the processing of gamma for intermediate theta values. We show a more detailed depiction of this in new Supplemental Figure 10, where you can see the appearance of the underlying alpha driven spikes and the issues they cause for intermediate theta frequencies (5.5-7.5Hz).

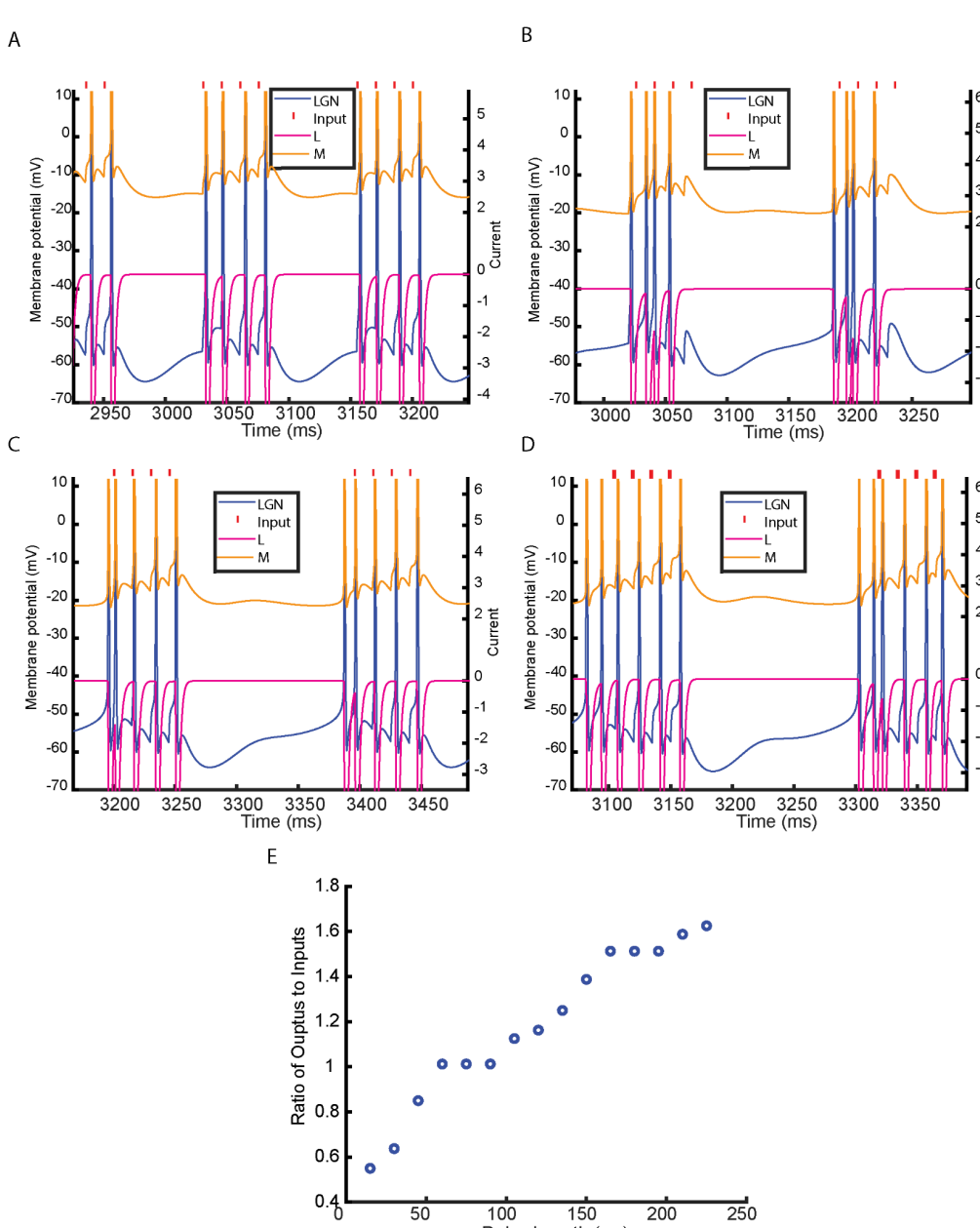

Supplemental Figure 10: Different theta timescales interact with the underlying alpha dynamics (produced by M and L current time constants) to determine passage of gamma inputs. The response of the TC cell to theta-gamma nested excitatory inputs depends on the incoming theta frequency. A-D) Response of the neuron to a nested theta-gamma excitatory signal where the gamma is at 66Hz for all plots and the theta is at 8, 6.5 ,5.1 and 4.5hz for A-D respectively. The gamma input is faithfully transmitted in A, C and D but there are additional spikes coming from the underlying alpha dynamics appearing in the TC output. The faithful transmission of gamma input is disrupted in B where the underlying alpha spikes are appearing in the middle of the gamma timed inputs. E) Summary plot showing the ratio of total input spikes to output spikes as a function of different theta timescales with a fixed incoming gamma frequency (66hz) and a fixed number of incoming spikes (4). Notably, the ratio of input to output spikes increases as the delay length between gamma bursts is increased due to the interaction with the underlying alpha rhythm.

We write in section 3.6 why the model separates into two distinct theta sub-bands:

We find that entrainment success depends on the nested frequencies of the input signal as well as the phase alignment between the underlying dynamics and incoming signals, a feature controlled in state IV by the interactions of the M current and L-type calcium current (Supplemental Figure 10).

6. Does the model provide insight into the mechanisms of thalamocortical slow oscillations (delta band or lower)?

We have added two new supplemental Figures to show how the model reproduces these key thalamic oscillations. These new Figures are supplemental Figures 1 and 4 which are provided above with their accompanying descriptive text in response to the first reviewer's comments and critiques about Figure 1 and and Figure 2 (at the start of reviewer 1's reviews). We provide here the associated text from section 2.2 that describes how the model transitions in and out of the low-threshold bursting states (I and II) from/out of the awake states (III,IV, and V) here:

Figure 2 also shows the impact of modulating gkl if external excitation (Iapp) is kept constant (orange vertical dashed lines). Starting in any of the awake states (III,IV,V), for any fixed value of Iapp, increasing gkl from 0 brings the neuron to a lower state of arousal. In spite of this, one subtle observation is that, with fixed excitation, the neuron cannot transition through the full range of states purely with gkl manipulation (Figure 2 orange lines). While modulation of gkl is sufficient to transition the thalamus between light sleep and resting awake states, reaching the slowest low-threshold bursting frequencies (< 1Hz) associated with slow wave sleep (found at the left edge of region I in the model), from any of the awake states (III,IV,V), requires additional changes in external thalamocortical cell input (Supplemental Figure 4) [42].

7. Some of the oscillation entrainment results resonate with similar phenomena in hippocampal and neocortical regions. Could there be similar filtering properties in the background? It might be interesting to discuss.

We thank the reviewer for making this connection, and now expand upon this idea and the relationship of our work with the hippocampal/neocortical experimental and modeling literature in discussion section 3.6 following our discussion on how our model relates to the thalamocortical dysrhythmia literature on theta-gamma. We discuss this connection as a part of our new larger discussion point on theta-gamma entrainment and its functional significance. We relate our work to prior research both in the hippocampus and other thalamic nuclei. We also discuss how the underlying dynamics and M and L currents in the model are instrumental in determining theta gamma passage properties. The entire new discussion paragraph on theta-gamma is provided below but we have bolded specifically the components below that addresses the reviewers comment about the hippocampus:

Outside of their relationships with TCDs (thalamocortical dysrhythmias), experimental work within and outside of the thalamus suggests that gamma nested slow oscillations may play an important role in thalamic communication and coordination with other brain areas. Theta-gamma oscillations are most prominently studied in the hippocampus for their positive relationships between cross-frequency-coupling and memory recall [98, 99]. In addition, recent work has shown evidence that theta-gamma coupling in the anterior thalamic nucleus is important for memory encoding [100], while pulvinar alpha can be coupled to cortical gamma influencing network synchrony and attentional selection [101]. Our modeling results suggest that gamma-nested slow oscillations (theta/alpha) may be possible within multiple thalamic nuclei, and that successful entrainment is likely dependent on the initial dynamic behavioral state. We find entrainment success depends on the nested frequencies of the input signal as well as the phase alignment between the underlying dynamics and incoming signals, a feature controlled in State IV by the interactions of the M and L-type calcium currents (Supplemental Figure 10). **Interestingly, these two currents are both expressed in brain areas such as the hippocampus and neocortex [32, 33], areas where theta-gamma coupling is prominent and has been attributed to properties of the M-current [102]. In addition to the theta-gamma coupling, in our model the interactions of the M and L-type calcium currents produce excitatory and inhibitory periodic entrainment in the alpha and beta bands; future studies could examine whether the interactions of these expressed currents in the hippocampus and neocortex might display similar results.**

Disclaimer: The modeling equations look right to me, but assessing the validity of all biophysical parameters in supplemental methods is beyond my expertise.

reviewer #3: Major
1. Slow 1 Hz oscillations and cholinergic agonism.
The slow ~1 Hz oscillations in the in vitro preparation described in ref. 28 were obtained without cholinergic agonists, whereas the manuscript appears to associate these slow oscillations with cholinergic modulation. The cited experimental work states:
"Experimental studies (discussed in detail in the Methods) show that with increasing depolarization via an applied current, in the presence of a cholinergic agonist, a thalamocortical cell produces five distinct dynamic firing types [22, 28, 29]"
"Furthermore, the bistability of region II (combined with the increased width of region II for low ACh) could help explain why, prior to agonist application, experimentalists observed a quiescent state that was nonexistent after agonist application [28]"
As in Hughes et al. (2002, Neuron), the agonist used was trans-ACPD, a non-specific group I/II mGluR agonist, rather than a cholinergic compound. These studies do not support a direct link between cholinergic agonism and the slow ~1 Hz oscillations. Please revise the corresponding claims accordingly and discuss how hypothetical cholinergic modulation could generate 1 Hz slow waves in your model (firing dynamic I), since the experimental

references cited do not support the model claims in their current form. This point is important because previous models missed the slow tonic firing regime: "Experimentally, the full dynamic range (all five states) was observed only after application of either a cholinergic or glutamatergic agonist [29, 35]." As a experimentalist, I find it difficult to determine which components of the model are directly grounded in experimental data that support the stated claims. For example, ref. 21 corresponds to the Vijayan et al. model, yet it is cited together with experimental statements such as"Awake alpha rhythms have been observed repeatedly in numerous species, within multiple brain regions, and correlated with a variety of cognitive functions [21,34,78–80]." Please make the distinction more explicit.

We thank the reviewer for their detailed reading and their comprehensive understanding of the relevant literature. We agree with the reviewer that our description of the experimental literature and how our model aligns with it was not sufficiently clear. While some of our changes to address this issue have already been outlined in response to other reviewer comments, we provide a complete answer here for such a comprehensive major revision point.

We have modified the writing of results sections 2.1 and 2.2 within our paper to properly align with the experimental work. This includes being more clear about the experimental literature as well as adjusting the claims made by our model. We emphasize that the alterations of gkl taking place in the model could be due to glutamatergic signaling thus providing alignment with experimental studies, The relevant text in 2.1 is now:

"Experimental studies (discussed in detail in the Methods) show that by increasing depolarization via an applied current in the presence of a metabotropic glutamate receptor (mGluR1) agonist, a thalamocortical cell produces five distinct dynamic firing types [22, 28, 29]. These studies also reveal that at least one of these novel firing types, the high threshold bursting alpha rhythm, is exhibited in the presence of cholinergic (mACHr) agonists [30]

…

Our model produces five distinct dynamic firing types, including the elusive experimentally observed high-threshold alpha-frequency bursting state [30, 35]. The full dynamic range in the model can be found by decreasing the background potassium conductances, an action used in computational modeling to mimic either an applied cholinergic or glutamatergic agonist effect [18, 21, 31], and then continually increasing Iapp in order to depolarize the cell.

…

While the five states in Figure 1 were found under fixed ionic conductance levels by varying the external excitation Iapp, experimental work has shown some of these state transitions occur in

the presence of neuromodulation [40–43]. Specifically, glutamate (Glu) and acetylcholine (ACh) are implicated in the transition from sleep to waking states via their respective activation of metabotropic glutamate receptors (mGluRs) and muscarinic acetylcholine receptors (mAChRs) [41, 43–47]."

In section 2.2 we provide further clarification about how our model interfaces with the neuromodulation literature. We highlight that in the model the manipulation of the ionic conductances gkl and gM could be due to either type (Ach or glutamate) of neuromodulatory signal. We therefore do not make claims about which neuromodulator effect our model is replicating, and instead frame section 2.2 as predictions about what would happen when a neuromodulator alters the potassium channel conductance. The relevant text is:

It is well documented that glutamatergic signals from the cortex and cholinergic signals from the brainstem play a strong role in transitioning the thalamus from sleep states to wakeful states [42, 44–48]. These arousal signals are typically instantiated in conductance-based thalamic models by decreasing the background potassium channel conductances of Ikl or IM [18, 35, 43, 49]. This action replicates one (of many) downstream effects of glutamate and acetylcholine via distinct signaling pathways involving mGluR and mAChR receptor (both expressed throughout the thalamus) activation [32, 33, 44, 50–53]. Depending on receptor and ion channel expression levels these two neuromodulatory signals may alter one, or both, of Ikl or IM to varying degrees; for simplicity, moving forward we refer not to the specific neuromodulator but to the modified ionic current.

Each of the below sections of text from 2.2 are where we have changed from referring to a particular neuromodulator to instead referring to the modulation of gkl.

Experimentally, the full dynamic range was observed in slice experiments only after application of a glutamatergic agonist [29, 30]. Without glutamatergic agonist application, experimentalists observed a smaller number of distinct dynamic states [28]. The model could help explain this experimental observation with the central plot of Figure 2 by considering two levels of modulated gkl (proposed conductance altered by the applied agonist) and varying Iapp across an identical fixed range (blue horizontal dashed lines).

…

These results indicate that neuromodulatory changes of gkl could increase the total number of possible observed dynamic states, while also impacting which state transitions are possible.

Figure 2 also shows the impact of modulating gkl if external excitation (Iapp) is kept constant (orange vertical dashed lines).

…

While modulation of gkl is sufficient to transition the thalamus between light sleep and resting awake states, reaching the slowest low-threshold bursting frequencies (< 1Hz) associated with slow wave sleep (found at the left edge of region I in the model), from any of the awake states (III,IV,V), requires additional changes in external thalamocortical cell input (Supplemental Figure 4) [42].

Finally we have gone through our citations to properly reference experimental papers for experimental findings and modeling papers for modeling findings.

2. The role of cortical alpha in V1.

In McGahan's model, alpha activity is described as a thalamic "working state" that emerges intrinsically from standard thalamocortical cells under cholinergic modulation. Traub et al. (2020), however, report alpha-frequency activity generated in neocortical slices in the absence of thalamic input, and propose that the classical eyes-closed visual alpha rhythm originates primarily in neocortical layer 4. Under their experimental conditions, alpha does not require the thalamic state described by McGahan to manifest, suggesting that neocortical circuitry is sufficient to generate alpha-band oscillations.

Given these results, it would be helpful to comment on how thalamic (LGN) and cortical (V1) alpha-generating mechanisms might interact, particularly since Traub et al. attribute alpha activity to dendritic bursting in layer 4 pyramidal neurons mediated by NR2C/D-containing NMDA receptors, which lack the conventional magnesium block, together with subthreshold EAG potassium conductances (Kv10.2). Clarifying whether these mechanisms are expected to coexist in a single model, dominate under different conditions, or interact dynamically would strengthen the interpretation of your simulations.

As the reviewer says, the mechanism for this form of cortical alpha is very different from thalamic alpha. Notably there are pathways from V1 to the thalamus and from the thalamus to V1 that could allow for cortical alpha to influence the thalamic alpha and vice versa. We show in our model that how the thalamus handles/entrains to the cortical alpha will depend on the properties of the cortical alpha rhythm (more or less spikes per cycle). We find that the thalamus entrains to the alpha less successfully when there are more spikes in the alpha frequency input. We show below the updated supplemental Figure that is discussed in section 3.5:

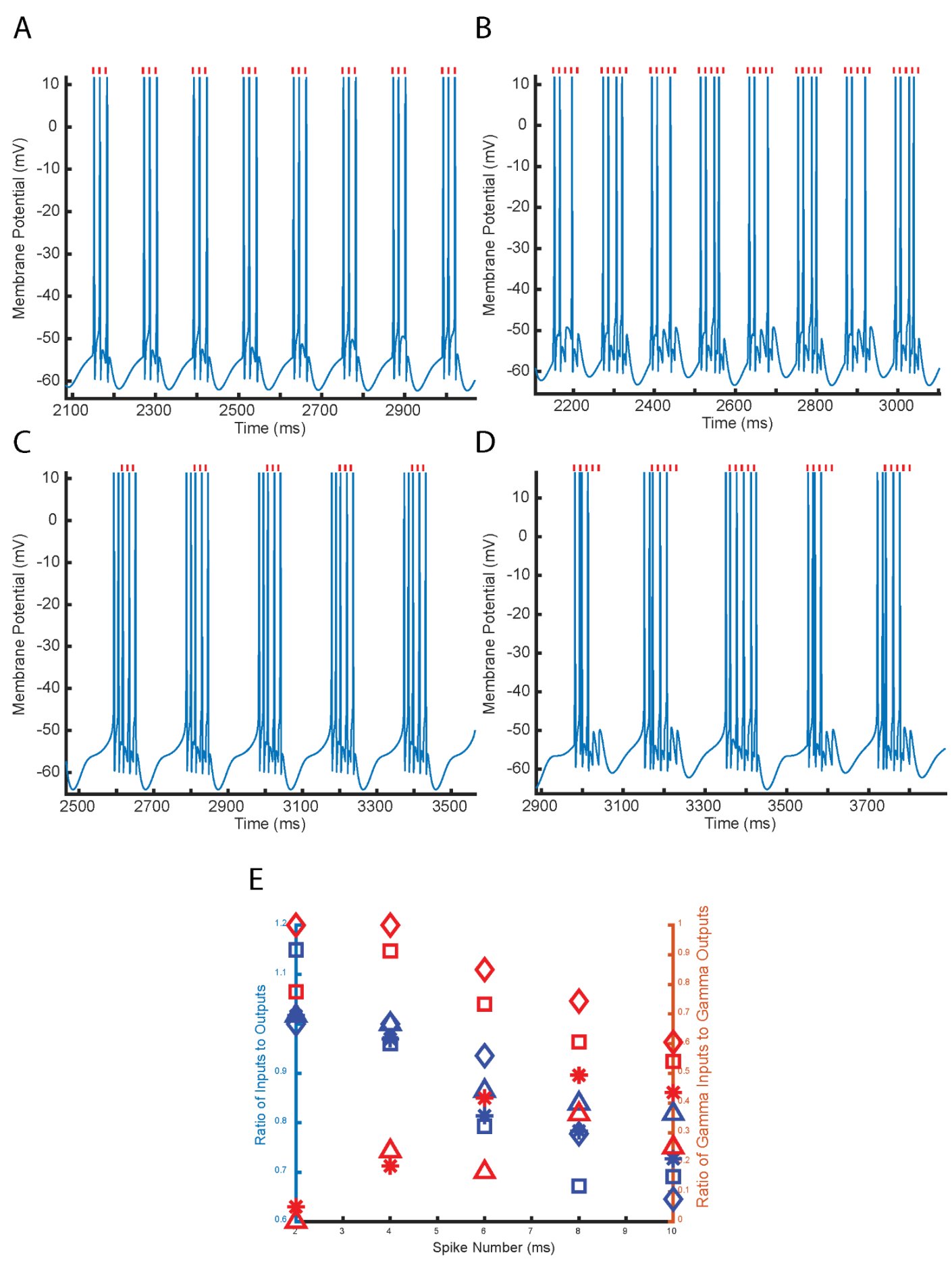

Supplemental Figure 11: Successful response of the TC cell to incoming nested excitatory gamma rhythms also depends on the number and frequency of the incoming gamma spikes. A) and B) Sample membrane potential trace of the neuron in state IV responding to 3 (A) or 5 (B) incoming gamma (66hz or 15ms ISI) spikes per alpha (8hz) cycle. The thalamus successfully passes all gamma spikes with 3 input spikes but fails to generate 100 percent passage of spiking for 5 spiking inputs. Red tick marks denote the timing of the input spikes. C) and D) Sample membrane potential trace of the neuron in state IV responding to 3 (C) or 5 (D) incoming gamma (66hz or 15ms ISI) spikes per theta (5hz) cycle. The thalamus successfully passes all gamma spikes with 3 input spikes but fails to generate 100 percent passage of spiking for 5 spiking inputs. Red tick marks denote the timing of the input spikes. Red tick marks denote the timing of the input spikes. E) Summary plot describing the relationship between the gamma spikes generated by the thalamus (in state IV) in response to different spike numbers and frequencies. Note the delay length (90ms) here is fixed for all simulations. Blue symbols correspond to the left axis which showcases the ratio of input spikes to output spikes regardless of frequency. Red Symbols correspond to the right axis and denote the ratio of incoming gamma spikes to outgoing gamma spikes at a similar (±3ms ISI) gamma frequency. Different symbol shapes correspond to different speeds of gamma inputs as follows: Triangle 25ms ISI, Asterisk 20ms ISI, Square 15ms ISI, and Diamond 10ms ISI. This Figure indicates that increasing the number of spikes per slow cycle, or decreasing the input gamma frequency, decreases the reliability of gamma transmission. In fact, for slower gamma inputs (50Hz or 40Hz), regardless of the number of input spikes per slow cycle, less than fifty percent of the output spikes occur at a matching gamma frequency. As a final note, for all input frequencies tested, fewer spikes per slow cycle helps maximize the total (irrespective of frequency) percent transmission of inputs. Together these results suggest that faster gamma frequencies with fewer incoming spikes per slow cycle, are likely to be relayed better.

In section 3.5 we hypothesize (and discuss) how cortical and thalamic alpha might interact based on our results of Supplemental Figure 11. This includes discussing how the thalamus might handle incoming cortical alpha and how V1 might handle incoming thalamic alpha. We speculate on some of these interactions here:

The awake alpha rhythm was the first recorded human brain rhythm and has been observed repeatedly in numerous species [36, 92–94]. The origins of the awake alpha rhythm are still hotly debated. Some studies have attributed the generation of alpha to the cortex [23, 95], others relate awake alpha power to distinct nuclei of the thalamus (such as the LGN) [36], and others consider alpha to arise as an interaction of the two [21, 96]. It remains an open question if and how these mechanisms interact [23]. Our model predicts that in the absence of external inputs the thalamus generates a clear alpha that may be influential in driving a cortical V1 alpha. We also predict that the thalamus will entrain to corticothalamic feedback at alpha frequencies depending on the shape (number of spikes per alpha rhythm peak) of the alpha input (Supplemental Figure 11). In tandem, these predictions suggest a context and signal dependent coexistence of the thalamic and cortical alpha rhythms that could be tested in a future cortico-thalamo-cortical model.

3. Functional role of alpha.
The manuscript states:
"We propose a new understanding of the thalamic alpha rhythm: this rhythm serves as a signal to the brain that the thalamus is in a working state prepared to filter both rhythmic and non-rhythmic incoming input; the alpha rhythm itself is not explicitly processing, or suppressing, information through the thalamus."
From an experimental perspective, it remains unclear how readers can disentangle whether alpha is epiphenomenal or has a causal role. Does the model generate any predictions that could distinguish these possibilities? Any interpretation will satisfy the reviewer but I find this important point needs to be more explicit (see van Bree et al., 2025 and Zhang et al 2022 for optogenetic stimulation of pyramidal cells in parietal cortex).

This question from the reviewer led to an important new way of describing our work. The essential point is that the M and L current are producing the filtering and entrainment properties and the alpha is just the output from those currents in the absence of noise. Thus, it is not the "alpha" that produces the filtering, but the M and L currents that produce both the alpha and filtering. This has clear experimental implications, since the M and L currents can be manipulated. Note although there are some similarities in filtering properties and entrainment properties between states III and IV, state IV is where the baseline high-threshold bursting frequency is actually occurring at alpha. Thus when we are referring to functional processes occurring during alpha, we are considering the model to be in state IV. We now emphasize our ideas about alpha in the introduction:

Our model results lead to new understanding of the awake thalamic alpha rhythm: the rhythm serves as a marker that the thalamus is in a working state. The associated filtering and entrainment capabilities that have been previously associated with changes in the alpha rhythm are dictated by interactions of the M current and L-type calcium current which happen to produce an alpha rhythm in the absence of input.

We then summarize our understanding of the thalamic alpha (which occurs in State IV) in discussion section 3.5.

Functionally, alpha rhythms have been hypothesized to: act as an idling state, increase in power with memory load or memory maintenance, exhibit elevated power during task distractor stimuli presentation, have a causal role in the allocation of spatial attention, and be inversely correlated with neuronal spiking and gamma band activity [92, 97 –101]. Here we propose a new functional understanding (consistent with the above interpretations) of the thalamic alpha rhythm: this rhythm is a marker that the thalamus is in a working state prepared to filter both rhythmic and non-rhythmic incoming input; the alpha rhythm itself is not explicitly processing, or suppressing, information through the thalamus. Instead, the model state IV (where the baseline rhythm is alpha) is where IM and ICaL are interacting to produce filtering and entrainment capabilities; it happens that in the absence of external signals this combination is producing an alpha.

Our model has the following important properties: it produces alpha in the absence of inputs, it exhibits decreasing alpha in response to sensory and non-alpha-frequency rhythmic signals, and it entrains to periodic alpha frequency inputs. These properties are in agreement with experimental observations and can be used to provide mechanistic explanations. Examples are: why the alpha rhythm is largest during periods of eyes closed relaxed wakefulness and is sharply decreased upon eyes opening [102] (an absence of sensory inputs into our model increases alpha); why alpha amplitude decreases when attending to a stimulus [22, 103] (an increase of external inputs to our model decreases alpha); and why alpha is increased during memory maintenance [104] (a top down cortical-produced alpha seen during memory maintenance entrains thalamic output in our model thereby increasing alpha power).

In section 3.5 we include a testable prediction that varying M and L currents will disrupt the production of alpha and will change the filtering properties in the thalamus accordingly. This is in the text:

We therefore predict that manipulations of the conductances and kinetic properties of IM and ICaL that disrupt the baseline alpha (or beta) rhythms will also disrupt many of these functional properties currently attributed to these rhythms as well.

Note, our model also makes testable predictions about how modulating the L-type current in manners consistent with schizophrenia literature may alter thalamic rhythmic processing and underlying frequencies. This is described above in response to reviewer 1's comments about exploring the novel components of the model and are pertaining to the new result section 2.8 in the manuscript.

The manuscript further states:
"The model predicts that the alpha rhythm will disappear in response to increases in visual stimulation, attention, or other possible rhythmic signals but, critically, the underlying dynamic state that produces the alpha rhythm in the absence of input remains the same.

Thus, if the thalamus is not receiving strong external input, the model can continue to produce a prominent alpha rhythm".
This framing suggests that event-related desynchronization (ERD) reflects a change in what patterns thalamic output rather than a transition to a different arousal state. Traub et al. (2020) describe a form of intracortical filtering associated with the layer 4 alpha rhythm. It would therefore be key to discuss whether the same dynamical state that produces thalamic alpha in the absence of input could also explain ERDs under strong stimulation, particularly given that post-stimulus alpha power appears to reach a floor (Iemi et al., 2019, Fig. 6C). Explicit computation of LFPs and ERDs would allow the model to generate predictions that are directly testable experimentally.

Our model shows that the thalamic alpha will go away in response to visual input (see response above to reviewer 3's questions about cortical alpha) that creates an ERD in cortex. To the extent that alpha in cortex is driven by the alpha in thalamus, that could explain the decrease in cortical alpha. As Traub shows, not all cortical alpha comes from the thalamus; however it is well accepted that there is a very large interaction between the thalamus and cortex in the alpha frequency range. The papers that you helpfully provided for us are evidence of this acceptance. We write in the text in 3.5:

Our model predicts that, in the absence of external inputs, the thalamus generates a clear alpha that may be influential in driving a cortical V1 alpha.

…

However in the presence of inputs that could lead to an ERD in cortex we write that the thalamus is:

it exhibits decreasing alpha in response to sensory and non-alpha-frequency rhythmic signals

Additionally, LFPs and ERDs represent signals from integrated synaptic input and dendritic processing calculated from multiple neurons at one time interacting with interneurons. A model involving multiple TC cells and TRN cells would be needed to fully answer the question of the reviewer; our model is a first step.

4. Update for current debates.
- Update literature on the functional role of alpha right now:
The functional role of alpha remains under active debate, particularly with respect to distraction suppression versus target processing (e.g., Gaspelin et al., 2023; Liesefeld et al., 2024; Jensen, 2024; Bonnefond et al., 2025; Keitel et al., 2025). The statement:
"Despite its ubiquity, the functional role of the awake alpha rhythm is hotly debated: specifically, does it serve to process task relevant information, filter out irrelevant information, or act as an idling state [78, 81]?"
would benefit from being updated in light of this recent literature.

We thank the reviewer for such an extensive list of useful sources and citations and have updated our discussion (and reference of the literatures) of the alpha rhythm accordingly. Our text introducing the functionality of alpha in discussion section 3.5 now states:

Functionally, alpha rhythms have been hypothesized to: act as an idling state, increase in power with memory load or memory maintenance, exhibit elevated power during task distractor stimuli presentation, have a causal role in the allocation of spatial attention, and be inversely correlated with neuronal spiking and gamma band activity [92, 97 –101].

In the text, we talk about how alpha interacts with different inputs and how this may explain some of the experimental observations. We show our model alpha produces alpha in the absence of inputs, has decreased alpha in response to inputs, and entrains to rhythmic alpha inputs. We describe these interactions and relationships with experimental observations in section 3.5. This text is provided above in response to reviewer 3's comments on the Function Role of Alpha.

- White noise vs Gaussian noise.
Recent work has used white-noise optogenetic perturbations to dissect circuit dynamics (Lewis, Ni et al., 2021; Day-Cooney et al., 2022; Cone et al., 2024). In the gamma range, white-noise perturbations revealed that V1 preferentially transmits input components coherent with endogenous rhythms, with the directed transfer function shaped by the M-current. In line with your suggestions, it would be valuable to relate these results to alpha, particularly since retinal ganglion cells, LGN, and V1 have long been studied using white-noise sequences (Chichilnisky, 2001; Ringach & Shapley, 2004), and white-noise luminance sequences in humans evoke strong alpha impulse responses in EEG (VanRullen et al., 2012), interpreted as traveling waves (Lozano-Soldevilla & VanRullen, 2019) with predictive-coding properties (Alamia & VanRullen, 2019).

Some preliminary simulation results suggest that the effect of adding white noise into our model depends on the strength of the noise. For a small amount of white noise, the model produces a slightly perturbed alpha-rhythmic output. However, as the strength of the white noise is increased, it acts similar to other inputs into our model by perturbing from the baseline alpha rhythm (see previous response above regarding the functional role of alpha).

Notably, directly increasing white noise in our LGN cell model and recording the LGN cell output could be functionally quite different from exposing the retina to a white noise luminance sequence and recording a whole brain EEG rhythm. The resulting strong alpha impulse responses in EEG mentioned by the reviewer (VanRullen et al., 2012) could be a combination of retinal, thalamic, and cortical response properties, which is beyond the scope of this project.

Minor
• Please add units to all color bars and explain them in the corresponding Figure captions (e.g., Figs. 4 and 6).

We have updated any Figures with color bars (and their corresponding captions) to include units when applicable. This includes Figures 4, 6, and the new Figure 10 (see above response to reviewer 1 regarding novel model components for included Figures). Note that Figures 4B, 4D, 6B, and 6D are probabilities meaning they are unitless. Figures 4C and 6C have had colorbars updated to be in ms with the caption describing:

"The delay time in milliseconds (colorbar value)"

The colorbars for all panels in Figure 10 are Frequencies in units of Hz which is described in the caption:

"All entrainment frequencies (given in Hz) are presented as color intensity (depicted on colorbar)"

• Clarify what the red circles represent (retinal spikes) and use consistent Figure conventions throughout (including Supplementary Fig. 3).

We have updated Figures to be consistent and to have clear meaning throughout. We also have changed the red circles to tick marks to align with common conventions and increase clarity.

• Although this is a single-cell model rather than a network, it would be helpful to speculate about potential phasing mechanism (Lorincz et al 2009, Vijayan et al 2012), particularly for regimes III and IV. Depending on whether interneurons operate in single-spike or burst mode, the TC cells of your model are forced to fire near the peak or trough of the alpha cycle; clarifying how your model accounts for this mechanism would strengthen the link to network-level phenomena.

We predict the network model in Lorincz 2009 and Vijayan 2012 that uses our version of a high-threshold bursting TC cell would still produce the same phasing of the other TC cells to the alpha rhythm. In the proposed Lorincz network model (which is replicated in the Vijayan computational modeling study) there are high threshold bursting neurons, producing an alpha that send inputs to interneurons that then phase the outputs of non-high-threshold bursting TC cells near the peak or trough of the alpha cycle. Thus the phasing stems from network interactions with the feedforward alpha. Notably, our model produces an identical looking alpha to previous high-threshold bursting models, even though the underlying dynamics and processing capabilities are different. The new text in section 3.8 is:

Previous network models of the thalamus have postulated that alpha-bursting TC cells can phase the outputs of non-bursting cells [30, 35]. Because the alpha bursting cells in these network models provide a driving alpha signal unaffected by feedback signals, we predict that the same results would emerge if our TC cells were used to produce the alpha in these network models instead. However, in general, these alpha producing TC cells are connected with gap junctions

and are receiving feedback inhibition (from the TRN and local interneurons) and feedback excitation (from the cortex). These interactions introduce additional time scales that filter or amplify a given signal in a manner distinct from the proposed processing of rhythmic signals we investigated in section 2.7 [3, 13, 29]. To fully explore the impacts of these network level effects would require a network structure.

• A recent study on pulvinar bursting statistics across idle and cognitive states (Boshra et al., 2025) appears relevant to Section 3.4 and could be discussed there.

We have made mention of the work by Boshra et. al. in our discussion about translating this model and its results to higher order thalamic nuclei. Specifically, this work by Boshra et. al., combined with the work by Ramcharan et. al. and Varela et. al., suggests that differences in neuromodulation for higher order versus lower order nuclei may indicate a change in the baseline resting awake processing state of higher order nuclei. We write in section 3.4:

This connection between low-threshold bursting and attention was recently examined directly in the pulvinar nucleus using electrical microstimulation; it was revealed that pulvinar bursting enhanced target detection and broadly increased control of cortical processing and behaviors [83]. This observed importance of low-threshold bursting in higher order cortical control may suggest that the baseline working state for higher order nuclei is state III (from which you can reach the low-threshold bursting state II more easily) and not state IV, as is proposed here for lower order nuclei.

• Alitto et al. (2025a) report that arousal, stimulus strength, and attention decrease beta power in LGN, whereas Alitto et al. (2025b) find that LGN spiking activity is weakly modulated, if at all. Commenting on how these observations fit within your model would clarify its predictions.

We have related the findings of Alitto et al.2025a to our work in the discussion section 3.5. Namely, we comment on how the decrease in baseline beta (defined in this case to be 11-35Hz) power in Alitto et. al. is similar to how our model exhibits a decreased alpha power in response to external stimuli and arousal levels. We suggest that these similarities between our alpha and the Alitto beta are the result of a similar mechanism where the rhythm is simply a marker for the underlying current interactions. We write in 3.5:

The model decrease in baseline alpha in response to external signals may have an analogue in the decreases of baseline beta (11-35Hz) power found in macaque [105]. Again, it may not be the frequency that is causal, but the interactions between the currents IM and ICaL (possibly with altered time constants or conductance values) that are causal. We therefore predict that manipulations of the conductances and kinetic properties of IM and ICaL that disrupt the

baseline alpha (or beta) rhythms will also disrupt many of these functional properties currently attributed to these rhythms as well.

The paper by Alitto et al (2025b) is very interesting to us, but its connection to our results is unclear. In our work, we describe the effects of inputs to the LGN under different arousal states determined by glutamatergic and cholinergic signaling as well as inputs from the retina. To the best of our knowledge, there is no work that connects the spatial attentional modulation discussed in Alitto et al (2025b) to the parameters we explore that describe different arousal states. To relate the 2025b paper to our work, we would need to know how spatial attention influences the rhythmic feedback signals from cortex and alters the retinal firing inputs.
