# Supplementary material for: Awake alpha bursting emerges as the dynamic working state in a lateral geniculate thalamocortical cell model
Source: PLoS Comput Biol. 2026 Oct 8;22(10):e1014654. doi: 10.1371/journal.pcbi.1014654 (PMC13649143; doi:10.1371/journal.pcbi.1014654)
Supplement: Supplementary file 3 [file pcbi.1014654.s020.docx]

Figure 1

Fig. 1I: The rhythm shown here appears to be around 5 Hz. Slow rhythms in thalamocortical (TC) neurons are typically < 1 Hz. Could the authors clarify how this frequency relates to experimentally observed slow rhythms?

Fig. 1II: The typical frequency of fast low-threshold bursting is often reported to be around 1 Hz, with a maximum of approximately 4 Hz in cat LGN slices. In contrast, the bursting activity here seems closer to 10 Hz. Unless I have overlooked something, these frequencies seem higher than those commonly observed in LGN slice recordings.

Even allowing for potential interspecies differences (rodent vs. cat vs. human), this discrepancy remains somewhat difficult to reconcile. Could the authors explain how an LTS can be generated at ~10 Hz in Fig. 1II? It may also be helpful to adjust the model so that its bursting dynamics are constrained within more physiological frequency ranges.

Finally, I suggest including a schematic of the model, with the ionic conductances and their distributions, to improve clarity and accessibility for readers.

Figure 2

This figure describes the dynamic states of the TC neuron model in the parameter space of Iapp and gKl. The results are consistent with Figure 1 (and therefore may reflect the same issue regarding frequency mismatch between the model output and experimental recordings).

In addition, the presentation could be improved. For example, showing transitions along a single row (or a defined trajectory in parameter space), and adding more intermediate examples, would help the reader better appreciate the *continuity* of the transitions between states.

Figure 3

This figure seems to present largely unprocessed simulation outputs. Some additional formatting and editing would greatly improve readability and interpretation.

In addition, the slow and delta frequencies remain higher than expected. It would be preferable to avoid notes on how to interpret the x-axis and instead present the data in a way that is immediately clear to the reader.

More generally, throughout the manuscript the authors frequently refer to specific experimental characteristics (e.g., p.11, line 199; and Figures 1–3 at least). Since not all readers will be familiar with the experimental literature (notably work from the Crunelli group), it would be very helpful to include schematic experimental traces or representative recordings side-by-side with the corresponding model outputs. This would strengthen the link between the model and its biological grounding.

Figure 4

Fig. 4D may not be necessary. Its contour could potentially be drawn directly on Fig. 4C, which would reduce redundancy and simplify the presentation.

Figure 5

Fig. 5D: The colored ISIs are difficult to see and are not clearly defined in the legend. In addition, it is unclear whether these results are quantified. Providing clearer definitions and/or quantitative measures would help improve interpretability.

Figure 6

Fig. 6D appears largely redundant with Fig. 6B and could potentially be omitted or integrated.

Figure 7

It might be clearer to represent retinal input using a vertical tick mark, rather than circles, to improve readability and consistency with common conventions.

Figure 8

Fig. 8A and 8C are difficult to interpret in their current form.
Fig. 8B would benefit from clarification (e.g., improved labeling and explanation).
Fig. 8D resembles a scatter plot, but the meaning is not clear: additional explanation, interpretation, and the inclusion of a trend or quantitative analysis would help the reader understand what is being conveyed.

Figure 9

Whenever the authors refer to “bursts” in this figure (and elsewhere), it would be helpful to specify whether these are high-threshold bursts (low frequency) or low-threshold bursts (high frequency), as this distinction is not readily apparent from the figure.

Major revisions

The key contribution of the manuscript is the proposal of a new thalamocortical neuron model. However, at this stage, the manuscript would benefit from additional work in order to strengthen confidence in the model and improve its interpretability.

In particular:

1. The model does not appear to reproduce the delta rhythm (~1–4 Hz) as reported extensively in previous experimental and modeling studies. This point should be addressed directly.
2. The manuscript does not sufficiently explore the impact of the model’s “novel” components. Systematic analysis of how these components influence firing regimes and rhythmicity would significantly strengthen the work.

More broadly, many figures consist of example traces without sufficient generalization or quantification (e.g., frequency, amplitude, variability), and without parameter sensitivity analysis. Since neuronal models often rely on fine-tuning of key parameters, the manuscript would greatly benefit from including an analysis of the most influential parameters and an evaluation of the robustness of the reported behaviors.

Finally, although the manuscript includes many references to experimental literature, it remains difficult for the reader to assess how well the model reproduces the specific experimental phenomena claimed. The authors could strengthen this point by presenting representative experimental data or schematics side-by-side with model outputs, or by including explicit quantitative comparisons to the relevant literature.
