# Supplementary material for: Awake alpha bursting emerges as the dynamic working state in a lateral geniculate thalamocortical cell model
Source: PLoS Comput Biol. 2026 Oct 8;22(10):e1014654. doi: 10.1371/journal.pcbi.1014654 (PMC13649143; doi:10.1371/journal.pcbi.1014654)
Supplement: Peer review history

# Peer review: Decision Letter 0

**DOI:** 10.1371/journal.pcbi.1014654.r001

**Authors:** Thomas Serre, Magor L Lorincz

29 Jan 2026

Awake alpha bursting emerges as the dynamic working state in a lateral geniculate thalamocortical cell model

PLOS Computational Biology

Dear Dr. McGahan,

Thank you for submitting your manuscript to PLOS Computational Biology. After careful consideration, we feel that it has merit but does not fully meet PLOS Computational Biology's publication criteria as it currently stands. Therefore, we invite you to submit a revised version of the manuscript that addresses the points raised during the review process.

Please submit your revised manuscript by Mar 31 2026 11:59PM. If you will need more time than this to complete your revisions, please reply to this message or contact the journal office at ploscompbiol@plos.org. When you're ready to submit your revision, log on to https://www.editorialmanager.com/pcompbiol/ and select the 'Submissions Needing Revision' folder to locate your manuscript file.

Please include the following items when submitting your revised manuscript:

* A letter that responds to each point raised by the editor and reviewer(s). You should upload this letter as a separate file labeled 'Response to Reviewers'. This file does not need to include responses to formatting updates and technical items listed in the 'Journal Requirements' section below.

* A marked-up copy of your manuscript that highlights changes made to the original version. You should upload this as a separate file labeled 'Revised Manuscript with Track Changes'.

* An unmarked version of your revised paper without tracked changes. You should upload this as a separate file labeled 'Manuscript'.

If you would like to make changes to your financial disclosure, competing interests statement, or data availability statement, please make these updates within the submission form at the time of resubmission. Guidelines for resubmitting your figure files are available below the reviewer comments at the end of this letter

We look forward to receiving your revised manuscript.

Kind regards,

Magor L Lorincz

Guest Editor

PLOS Computational Biology

Thomas Serre

Section Editor

PLOS Computational Biology

**Additional Editor Comments:**

Dear Dr. McGahan and colleagues,

Thank you for submitting your manuscript, “Awake alpha bursting emerges as the dynamic working state in a lateral geniculate thalamocortical cell model,” to PLOS Computational Biology. We have now received and carefully considered three detailed reviews.

All reviewers recognize the novelty and potential impact of this work. In particular, the construction of a relatively compact, biophysically grounded thalamocortical cell model that unifies intrinsic firing regimes, neuromodulation, sensory filtering, and rhythmic entrainment is viewed as a significant contribution. The proposed roles of the M-current and L-type calcium current in awake-state filtering and rhythmic processing are especially compelling, and the manuscript has the potential to become an important reference for future thalamocortical modeling studies.

At the same time, the reviewers agree that substantial revisions are required before the manuscript can be considered for publication. Several core claims are currently overstated or insufficiently supported, and key aspects of biological grounding, validation, and interpretability need to be strengthened. For these reasons, we invite you to submit a major revision addressing the points below.

The manuscript repeatedly associates slow (~1 Hz) oscillations and state transitions with cholinergic modulation. However, the experimental literature cited (e.g., Hughes et al., Crunelli et al.) does not support a direct causal link between cholinergic agonism and the slow oscillatory regime, as these dynamics were observed in the absence of cholinergic compounds and involved mGluR agonists. While in cortical in vitro preparations reinstating the cholinergic tone revealed a mostly synaptically generated slow oscillation (Lorincz…Crunelli, 2016 JN), in thalamic slices the cholinergic tone led to alpha bursts (Lorincz et al 2008, 2009) but not to slow oscillation even if the cells were hyperpolarized by – d.c. Please revise the text to clearly distinguish experimentally established findings from modeling assumptions or hypotheses. Also correct or qualify claims that directly attribute slow oscillations to cholinergic modulation. Explicitly state when cholinergic effects are hypothetical or indirect within the model framework.

Several reviewers raise concerns that the frequencies of slow oscillations and low-threshold bursting in the model appear higher than those typically reported in LGN slice recordings. Because the manuscript’s central claim is that the model reproduces the full range of experimentally observed firing regimes, this issue must be addressed directly.

Please provide quantitative measurements (e.g., frequencies, burst rates, inter-burst intervals) for each firing regime, compare these values explicitly to known experimental ranges. Also either reconcile apparent discrepancies (e.g., species or preparation differences) or adjust model parameters and/or claims accordingly.

While the manuscript frequently states that the model reproduces experimental observations, this correspondence is not always demonstrated clearly. Please include representative experimental traces or schematic depictions (where feasible) alongside model outputs for key firing regimes or provide clearer quantitative comparisons to experimental results (e.g., ranges of frequencies, transmission probabilities, delay times).

To strengthen confidence in the generality of the findings please provide a more systematic analysis of the contribution of the model’s novel components (particularly IM and ICaL), such as ablation or sensitivity analyses. Address parameter robustness and clarify whether key behaviors require fine tuning. Improve figure clarity and presentation, reducing reliance on unprocessed example traces and adding clearer labels, units, and legends where needed. Consider including a schematic of the model summarizing ionic conductances and their interactions.

**Journal Requirements:**

1) We ask that a manuscript source file is provided at Revision. Please upload your manuscript file as a .doc, .docx, .rtf or .tex. If you are providing a .tex file, please upload it under the item type u2018LaTeX Source Fileu2019 and leave your .pdf version as the item type u2018Manuscriptu2019.

2) Please upload all main figures as separate Figure files in .tif or .eps format. For more information about how to convert and format your figure files please see our guidelines:

https://journals.plos.org/ploscompbiol/s/figures

3) Please amend your detailed Financial Disclosure statement. This is published with the article. It must therefore be completed in full sentences and contain the exact wording you wish to be published.

- State the initials, alongside each funding source, of each author to receive each grant. For example: "This work was supported by the National Institutes of Health (####### to AM; ###### to CJ) and the National Science Foundation (###### to AM)."

- State what role the funders took in the study. If the funders had no role in your study, please state: "The funders had no role in study design, data collection and analysis, decision to publish, or preparation of the manuscript.".

If you did not receive any funding for this study, please simply state: u201cThe authors received no specific funding for this work.u201d

**Reviewers' comments:**

Reviewer's Responses to Questions

**Comments to the Authors:**

**Please note here if the review is uploaded as an attachment.**

Reviewer #1: The reviewe is uploaded in the attachment.

Reviewer #2: McGahan et al. present a novel LGN thalamocortical cell model. It has a number of strengths:

1. It reproduces diverse experimentally observed firing patterns, providing insights into potential underlying mechanisms of state transitions at the ion channel level.

2. It provides insights into how retinal input is processed (blocked, filtered or faithfully transmitted) by the LGN in a state-dependent manner.

3. It links thalamocortical processing to neuromodulatory functions (primarily cholinergic).

4. It establishes possible mechanisms of oscillation entrainment in the thalamus in a frequency-specific manner, including the testing of nested oscillations.

The manuscript is a thorough and important piece that brings seminal electrophysiology work from the Crunelli lab and recent genetic data together in one relatively simple model that nevertheless produces complex dynamics. I think this will be an important reference point for thalamocortical modeling and information processing in the future. I only have a few points aiming to provide further insights as well as ease the understanding of the manuscript for the reader.

1. It would greatly increase the appeal of the model if the Authors could provide a direct demonstration of how the model reproduces experimentally observed dynamics, e.g. in Figure 1, even if only by replotting previous experimental results.

2. It is stated multiple times that the channel constitution is based on recent genetic data (e.g. from Allen Brain Institute). However, to make this claim clearer, more details are needed on how exactly the genetic data informed the model, preferentially even demonstrated visually.

3. There is some ambiguity in focusing on states III and IV vs. only on state IV. For instance, Fig. 5 could show both III and IV, given that the corresponding text claims they are similar, suggested also by the title of section 2.5.

4. The different role of the T-type Ca channels compared to previous models is a striking feature of the model that would deserve more focus. It is contemplated in the methods section 5.1, but maybe that discussion would more belong to intro/discussion sections. Is this difference rooted in the genetic data that were considered, and if yes, how?

5. The separation of theta sub-bands in Fig.8D is interesting. What could underlie this in the model?

6. Does the model provide insight into the mechanisms of thalamocortical slow oscillations (delta band or lower)?

7. Some of the oscillation entrainment results resonate with similar phenomena in hippocampal and neocortical regions. Could there be similar filtering properties in the background? It might be interesting to discuss.

Disclaimer: The modeling equations look right to me, but assessing the validity of all biophysical parameters in supplemental methods is beyond my expertise.

I believe that openness and transparency increase the fairness of peer review. Therefore, I am signing my reviews.

Balazs Hangya

Reviewer #3: Major

1. Slow 1 Hz oscillations and cholinergic agonism.

The slow ~1 Hz oscillations in the in vitro preparation described in ref. 28 were obtained without cholinergic agonists, whereas the manuscript appears to associate these slow oscillations with cholinergic modulation. The cited experimental work states:

"Experimental studies (discussed in detail in the Methods) show that with increasing depolarization via an applied current, in the presence of a cholinergic agonist, a thalamocortical cell produces five distinct dynamic firing types [22, 28, 29]"

"Furthermore, the bistability of region II (combined with the increased width of region II for low ACh) could help explain why, prior to agonist application, experimentalists observed a quiescent state that was nonexistent after agonist application [28]"

As in Hughes et al. (2002, Neuron), the agonist used was trans-ACPD, a non-specific group I/II mGluR agonist, rather than a cholinergic compound. These studies do not support a direct link between cholinergic agonism and the slow ~1 Hz oscillations. Please revise the corresponding claims accordingly and discuss how hypothetical cholinergic modulation could generate 1 Hz slow waves in your model (firing dynamic I), since the experimental references cited do not support the model claims in their current form. This point is important because previous models missed the slow tonic firing regime:

"Experimentally, the full dynamic range (all five states) was observed only after application of either a cholinergic or glutamatergic agonist [29, 35]."

As a experimentalist, I find it difficult to determine which components of the model are directly grounded in experimental data that support the stated claims. For example, ref. 21 corresponds to the Vijayan et al. model, yet it is cited together with experimental statements such as

"Awake alpha rhythms have been observed repeatedly in numerous species, within multiple brain regions, and correlated with a variety of cognitive functions [21,34,78–80]."

Please make the distinction more explicit.

2. The role of cortical alpha in V1.

In McGahan’s model, alpha activity is described as a thalamic “working state” that emerges intrinsically from standard thalamocortical cells under cholinergic modulation. Traub et al. (2020), however, report alpha-frequency activity generated in neocortical slices in the absence of thalamic input, and propose that the classical eyes-closed visual alpha rhythm originates primarily in neocortical layer 4. Under their experimental conditions, alpha does not require the thalamic state described by McGahan to manifest, suggesting that neocortical circuitry is sufficient to generate alpha-band oscillations.

Given these results, it would be helpful to comment on how thalamic (LGN) and cortical (V1) alpha-generating mechanisms might interact, particularly since Traub et al. attribute alpha activity to dendritic bursting in layer 4 pyramidal neurons mediated by NR2C/D-containing NMDA receptors, which lack the conventional magnesium block, together with subthreshold EAG potassium conductances (Kv10.2). Clarifying whether these mechanisms are expected to coexist in a single model, dominate under different conditions, or interact dynamically would strengthen the interpretation of your simulations.

3. Functional role of alpha.

The manuscript states:

"We propose a new understanding of the thalamic alpha rhythm: this rhythm serves as a signal to the brain that the thalamus is in a working state prepared to filter both rhythmic and non-rhythmic incoming input; the alpha rhythm itself is not explicitly processing, or suppressing, information through the thalamus."

From an experimental perspective, it remains unclear how readers can disentangle whether alpha is epiphenomenal or has a causal role. Does the model generate any predictions that could distinguish these possibilities? Any interpretation will satisfy the reviewer but I find this important point needs to be more explicit (see van Bree et al., 2025 and Zhang et al 2022 for optogenetic stimulation of pyramidal cells in parietal cortex).

The manuscript further states:

"The model predicts that the alpha rhythm will disappear in response to increases in visual stimulation, attention, or other possible rhythmic signals but, critically, the underlying dynamic state that produces the alpha rhythm in the absence of input remains the same. Thus, if the thalamus is not receiving strong external input, the model can continue to produce a prominent alpha rhythm".

This framing suggests that event-related desynchronization (ERD) reflects a change in what patterns thalamic output rather than a transition to a different arousal state. Traub et al. (2020) describe a form of intracortical filtering associated with the layer 4 alpha rhythm. It would therefore be key to discuss whether the same dynamical state that produces thalamic alpha in the absence of input could also explain ERDs under strong stimulation, particularly given that post-stimulus alpha power appears to reach a floor (Iemi et al., 2019, Fig. 6C). Explicit computation of LFPs and ERDs would allow the model to generate predictions that are directly testable experimentally.

4. Update for current debates.

- Update literature on the functional role of alpha right now:

The functional role of alpha remains under active debate, particularly with respect to distraction suppression versus target processing (e.g., Gaspelin et al., 2023; Liesefeld et al., 2024; Jensen, 2024; Bonnefond et al., 2025; Keitel et al., 2025). The statement:

"Despite its ubiquity, the functional role of the awake alpha rhythm is hotly debated: specifically, does it serve to process task relevant information, filter out irrelevant information, or act as an idling state [78, 81]?"

would benefit from being updated in light of this recent literature.

- White noise vs Gaussian noise.

Recent work has used white-noise optogenetic perturbations to dissect circuit dynamics (Lewis, Ni et al., 2021; Day-Cooney et al., 2022; Cone et al., 2024). In the gamma range, white-noise perturbations revealed that V1 preferentially transmits input components coherent with endogenous rhythms, with the directed transfer function shaped by the M-current. In line with your suggestions, it would be valuable to relate these results to alpha, particularly since retinal ganglion cells, LGN, and V1 have long been studied using white-noise sequences (Chichilnisky, 2001; Ringach & Shapley, 2004), and white-noise luminance sequences in humans evoke strong alpha impulse responses in EEG (VanRullen et al., 2012), interpreted as traveling waves (Lozano-Soldevilla & VanRullen, 2019) with predictive-coding properties (Alamia & VanRullen, 2019).

________________________________________

Minor

• Please add units to all color bars and explain them in the corresponding figure captions (e.g., Figs. 4 and 6).

• Clarify what the red circles represent (retinal spikes) and use consistent figure conventions throughout (including Supplementary Fig. 3).

• Although this is a single-cell model rather than a network, it would be helpful to speculate about potential phasing mechanism (Lorincz et al 2009, Vijayan et al 2012), particularly for regimes III and IV. Depending on whether interneurons operate in single-spike or burst mode, the TC cells of your model are forced to fire near the peak or trough of the alpha cycle; clarifying how your model accounts for this mechanism would strengthen the link to network-level phenomena.

• A recent study on pulvinar bursting statistics across idle and cognitive states (Boshra et al., 2025) appears relevant to Section 3.4 and could be discussed there.

• Alitto et al. (2025a) report that arousal, stimulus strength, and attention decrease beta power in LGN, whereas Alitto et al. (2025b) find that LGN spiking activity is weakly modulated, if at all. Commenting on how these observations fit within your model would clarify its predictions.

References:

Alamia, Andrea, and Rufin VanRullen. "Alpha oscillations and traveling waves: Signatures of predictive coding?." PLoS Biology 17, no. 10 (2019): e3000487.

Alitto, Henry J., Alyssa N. Sanchez, Prescot C. Alexander, and W. Martin Usrey. "Dynamic Modulation of Beta-Band Oscillations in the LGN and Their Role in Visual Processing." bioRxiv (2025a): 2025-07.

Alitto, Henry J., Jeffrey S. Johnson, and W. Martin Usrey. "Spatial Attention Weakly Modulates Visual Responses in the Lateral Geniculate Nucleus." bioRxiv (2025b): 2025-05.

Bonnefond, Mathilde, and Ole Jensen. "The role of alpha oscillations in resisting distraction." Trends in Cognitive Sciences 29, no. 4 (2025): 368-379.

Boshra, Rober, Michael Harris, Kacie Dougherty, Melissa Berg, Britney M. Morea, Henry J. Alitto, Mario C. Rodriguez, W. Martin Usrey, and Sabine Kastner. "Causal role for pulvinar burst firing in thalamo-cortical attention control." bioRxiv (2025).

Chichilnisky, E. J. "A simple white noise analysis of neuronal lightresponses." Network: computation in neural systems 12, no. 2 (2001): 199.

Cone, Jackson J., Autumn O. Mitchell, Rachel K. Parker, and John HR Maunsell. "Stimulus-dependent differences in cortical versus subcortical contributions to visual detection in mice." Current Biology 34, no. 9 (2024): 1940-1952.

Day-Cooney, Julian, Jackson J. Cone, and John HR Maunsell. "Perceptual weighting of V1 spikes revealed by optogenetic white noise stimulation." Journal of Neuroscience 42, no. 15 (2022): 3122-3132.

Gaspelin, Nicholas, Dominique Lamy, Howard E. Egeth, Heinrich R. Liesefeld, Dirk Kerzel, Ananya Mandal, Matthias M. Müller et al. "The distractor positivity component and the inhibition of distracting stimuli." Journal of cognitive neuroscience 35, no. 11 (2023): 1693-1715.

Jensen, Ole. "Distractor inhibition by alpha oscillations is controlled by an indirect mechanism governed by goal-relevant information." Communications Psychology 2, no. 1 (2024): 36.

Iemi, Luca, Niko A. Busch, Annamaria Laudini, Saskia Haegens, Jason Samaha, Arno Villringer, and Vadim V. Nikulin. "Multiple mechanisms link prestimulus neural oscillations to sensory responses." elife 8 (2019): e43620.

Keitel, Anne, Christian Keitel, Mohsen Alavash, Karin Bakardjian, Christopher SY Benwell, Sophie Bouton, Niko A. Busch et al. "Brain rhythms in cognition--controversies and future directions." arXiv preprint arXiv:2507.15639 (2025).

Lewis, Christopher Murphy, Jianguang Ni, Thomas Wunderle, Patrick Jendritza, Andreea Lazar, Ilka Diester, and Pascal Fries. "Cortical gamma-band resonance preferentially transmits coherent input." Cell reports 35, no. 5 (2021).

Liesefeld, Heinrich R., Dominique Lamy, Nicholas Gaspelin, Joy J. Geng, Dirk Kerzel, Jeffrey D. Schall, Harriet A. Allen et al. "Terms of debate: Consensus definitions to guide the scientific discourse on visual distraction." Attention, Perception, & Psychophysics 86, no. 5 (2024): 1445-1472.

Lozano-Soldevilla, Diego, and Rufin VanRullen. "The hidden spatial dimension of alpha: 10-Hz perceptual echoes propagate as periodic traveling waves in the human brain." Cell reports 26, no. 2 (2019): 374-380.

Ringach, Dario, and Robert Shapley. "Reverse correlation in neurophysiology." Cognitive Science 28, no. 2 (2004): 147-166.

Traub, Roger D., Karen Hawkins, Natalie E. Adams, Stephen P. Hall, Anna Simon, and Miles A. Whittington. "Layer 4 pyramidal neuron dendritic bursting underlies a post-stimulus visual cortical alpha rhythm." Communications biology 3, no. 1 (2020): 230.

van Bree, Sander, Daniel Levenstein, Matthew R. Krause, Bradley Voytek, and Richard Gao. "Processes and measurements: a framework for understanding neural oscillations in field potentials." Trends in cognitive sciences 29, no. 5 (2025): 448-466.

VanRullen, Rufin, and James SP Macdonald. "Perceptual echoes at 10 Hz in the human brain." Current biology 22, no. 11 (2012): 995-999.

Zhang, Mengsen, and Flavio Frohlich. "Cell type-specific excitability probed by optogenetic stimulation depends on the phase of the alpha oscillation." Brain stimulation 15, no. 2 (2022): 472-482.

**********

**Have the authors made all data and (if applicable) computational code underlying the findings in their manuscript fully available?**

The PLOS Data policy requires authors to make all data and code underlying the findings described in their manuscript fully available without restriction, with rare exception (please refer to the Data Availability Statement in the manuscript PDF file). The data and code should be provided as part of the manuscript or its supporting information, or deposited to a public repository. For example, in addition to summary statistics, the data points behind means, medians and variance measures should be available. If there are restrictions on publicly sharing data or code —e.g. participant privacy or use of data from a third party—those must be specified.

Reviewer #1: Yes

Reviewer #2: Yes

Reviewer #3: Yes

**********

PLOS authors have the option to publish the peer review history of their article (what does this mean?). If published, this will include your full peer review and any attached files.

If you choose “no”, your identity will remain anonymous but your review may still be made public.

**Do you want your identity to be public for this peer review?**  For information about this choice, including consent withdrawal, please see our Privacy Policy.

Reviewer #1: No

Reviewer #2: **Yes:**  Balazs Hangya

Reviewer #3: No

[NOTE: If reviewer comments were submitted as an attachment file, they will be attached to this email and accessible via the submission site. Please log into your account, locate the manuscript record, and check for the action link "View Attachments". If this link does not appear, there are no attachment files.]

**Figure resubmission:**

After uploading your figures to PLOS’s NAAS tool - https://ngplosjournals.pagemajik.ai/artanalysis, NAAS will process the files provided and display the results in the "Uploaded Files" section of the page as the processing is complete. If the uploaded figures meet our requirements (or NAAS is able to fix the files to meet our requirements), the figure will be marked as "fixed" above. If NAAS is unable to fix the files, a red "failed" label will appear above. When NAAS has confirmed that the figure files meet our requirements, please download the file via the download option, and include these NAAS processed figure files when submitting your revised manuscript.

**Reproducibility:**

?>

Attachment Submitted filename: Review.docx

# Author comment: Author response to Decision Letter 1

**DOI:** 10.1371/journal.pcbi.1014654.r002

9 Jun 2026

Attachment Submitted filename: Response to Reviewers.pdf

# Peer review: Decision Letter 1

**DOI:** 10.1371/journal.pcbi.1014654.r003

**Authors:** Thomas Serre, Magor L Lorincz

3 Aug 2026

Dear Dr McGahan,

We are pleased to inform you that your manuscript 'Awake alpha bursting emerges as the dynamic working state in a lateral geniculate thalamocortical cell model' has been provisionally accepted for publication in PLOS Computational Biology.

Before your manuscript can be formally accepted you will need to complete some formatting changes, which you will receive in a follow up email. A member of our team will be in touch with a set of requests.

Please note that your manuscript will not be scheduled for publication until you have made the required changes, so a swift response is appreciated.

IMPORTANT: The editorial review process is now complete. PLOS will only permit corrections to spelling, formatting or significant scientific errors from this point onwards. Requests for major changes, or any which affect the scientific understanding of your work, will cause delays to the publication date of your manuscript.

Should you, your institution's press office or the journal office choose to press release your paper, you will automatically be opted out of early publication. We ask that you notify us now if you or your institution is planning to press release the article. All press must be co-ordinated with PLOS.

Thank you again for supporting Open Access publishing; we are looking forward to publishing your work in PLOS Computational Biology.

Best regards,

Magor L Lorincz

Guest Editor

PLOS Computational Biology

Thomas Serre

Section Editor

PLOS Computational Biology

***********************************************************

Dear Authors,

Thank you for submitting the revised version of your manuscript.

I appreciate the substantial effort invested in addressing both the reviewers' comments and the editorial concerns. The major criticisms have been addressed successfully, and I believe the manuscript has been significantly improved as a result. The revisions have strengthened both the scientific rigor and the clarity of the work.

Before the manuscript can be accepted, I would ask you to address a few remaining minor points:

Please include a brief paragraph in the Discussion comparing your model with previously published thalamocortical neuron models (e.g., those by Bazhenov and colleagues, Hughes et al., Amarillo et al., David et al., Tissone et al., and Dervinis & Crunelli). This comparison will help readers better appreciate the specific strengths and novel contributions of the present model.

Please briefly acknowledge the remaining differences between the simulated membrane potential waveforms and experimentally observed thalamocortical physiology (e.g., the morphology of Up and Down states, plateau potentials, and afterhyperpolarizations). Naturally, no computational model is expected to reproduce every physiological detail, but a short discussion of these limitations would provide appropriate context.

Please update reference 92 to the published Neuron article, as noted by Reviewer 3.

Please clarify in the Figure 10 legend that the individual panels use different color scales, so readers do not inadvertently compare colors across panels quantitatively.

In the interest of transparency: bursts with similar properties have been recorded in vivo in the thalamus of lightly anesthetized mice (PMID: 21892727) and in awake mice (PMID: 34296165) — work from my own group, which is why I raise it only as a point of possible interest rather than a request. The authors should feel entirely free to disregard it.

Once these minor issues have been addressed, I will be pleased to accept the manuscript for publication.

Reviewer's Responses to Questions

**Comments to the Authors:**

**Please note here if the review is uploaded as an attachment.**

Reviewer #1: The revised manuscript provides a comprehensive overview of the possible dynamical behaviors of thalamocortical neurons. Overall, I believe that the manuscript has been substantially improved, and I recommend it for publication.

I have only a few minor comments:

1. It would be useful to compare the present model, at least qualitatively, with previously published cellular models of thalamocortical neurons, including those described in:

* Dervinis and Crunelli, CNS Neuroscience & Therapeutics (2024)

* Tissone et al., Journal of Neurophysiology (2021)

* Amarillo et al., Frontiers (2015) and Journal of Neurophysiology (2018)

* David et al., Frontiers (2016)

* Hughes et al., Neuron (2002, 2004)

* The various thalamocortical neuron models developed by Bazhenov and colleagues

Such a comparison would help readers better understand the specific strengths, limitations, and distinctive features of the proposed model.

2. A few aspects of the simulated activity do not fully reproduce the known physiology of thalamocortical neurons. For example, the model does not appear to really capture the characteristic shape of Up and Down states observed during slow oscillations, including the depolarized plateau near (-60) mV, as illustrated in Figures 1I and 2I. Similarly, the dynamics of the afterhyperpolarization following high-frequency bursts, as shown in Figure 3I, seem somewhat atypical, particularly the plateau near (-20) mV.

These discrepancies are understandable, as no model can reproduce every aspect of neuronal physiology. Nevertheless, they should be acknowledged and briefly discussed in the manuscript.

Reviewer #2: The Authors addressed my concerns.

I believe that openness and transparency increase the fairness of peer review. Therefore, I am signing my reviews.

Balazs Hangya

Reviewer #3: I congratulate the authors for their thorough work and efforts to reply to the issues I raised. After reading all of them, my concerns are resolved.

I thank the authors for the efficiency of their replies, given the high volume of high-quality extra figures they provided. The update of the claims, including the role of cholinergic and/or glutamatergic agonist effects, their link to experimental data, is now clear. The present model characterizes well-known roles of alpha (entrainment, filtering, resting) using two channels will definitely be food for thought for the cognitive neuroscience community.

I thank the authors for their reply regarding not including Alitto et al. 2025b. Modeling how spatial attention influences alpha and retinal input is beyond the scope of the paper. I thank the authors once again for educating us reviewers on these issues.

Lastly, I would like to raise awareness about to update ref 92 because the preprint has been recently accepted in Neuron.

Boshra, R., Harris, M., Dougherty, K., Berg, M., Morea, B. M., Alitto, H. J., … Kastner, S. (2026). Higher-order thalamic bursts are drivers of attention control. Neuron. doi:10.1016/j.neuron.2026.05.025

I would like to add a brief note to the caption in Figure 10 (entrainment) to warn the reader that the color code for each subplot has different scale: yellow in subplot A is about 40Hz but in C is about 16 Hz. The same story goes for B and D.

Congratulations once again for this sharing this very interesting piece.

**********

**Have the authors made all data and (if applicable) computational code underlying the findings in their manuscript fully available?**

The PLOS Data policy requires authors to make all data and code underlying the findings described in their manuscript fully available without restriction, with rare exception (please refer to the Data Availability Statement in the manuscript PDF file). The data and code should be provided as part of the manuscript or its supporting information, or deposited to a public repository. For example, in addition to summary statistics, the data points behind means, medians and variance measures should be available. If there are restrictions on publicly sharing data or code —e.g. participant privacy or use of data from a third party—those must be specified.

Reviewer #1: Yes

Reviewer #2: None

Reviewer #3: Yes

**********

PLOS authors have the option to publish the peer review history of their article (what does this mean?). If published, this will include your full peer review and any attached files.

If you choose “no”, your identity will remain anonymous but your review may still be made public.

**Do you want your identity to be public for this peer review?**  For information about this choice, including consent withdrawal, please see our Privacy Policy.

Reviewer #1: No

Reviewer #2: **Yes:**  Balazs Hangya

Reviewer #3: **Yes:**  Diego Lozano-Soldevilla

# Editor report: Acceptance letter

**DOI:** 10.1371/journal.pcbi.1014654.r004

**Authors:** Thomas Serre, Magor L Lorincz

PCOMPBIOL-D-25-02409R1

Awake alpha bursting emerges as the dynamic working state in a lateral geniculate thalamocortical cell model

Dear Dr McGahan,

I am pleased to inform you that your manuscript has been formally accepted for publication in PLOS Computational Biology. Your manuscript is now with our production department and you will be notified of the publication date in due course.

The corresponding author will soon be receiving a typeset proof for review, to ensure errors have not been introduced during production. Please review the PDF proof of your manuscript carefully, as this is the last chance to correct any errors. Please note that major changes, or those which affect the scientific understanding of the work, will likely cause delays to the publication date of your manuscript.

Soon after your final files are uploaded, unless you have opted out, the early version of your manuscript will be published online. The date of the early version will be your article's publication date. The final article will be published to the same URL, and all versions of the paper will be accessible to readers.

For Research, Software, and Methods articles, you will receive an invoice from PLOS for your publication fee after your manuscript has reached the completed accept phase. If you receive an email requesting payment before acceptance or for any other service, this may be a phishing scheme. Learn how to identify phishing emails and protect your accounts at https://explore.plos.org/phishing.

Thank you again for supporting PLOS Computational Biology and open-access publishing. We are looking forward to publishing your work!

With kind regards,

Janani Seenivasan

PLOS Computational Biology | Carlyle House, Carlyle Road, Cambridge CB4 3DN | United Kingdom ploscompbiol@plos.org | Phone +44 (0) 1223-442824 | ploscompbiol.org | @PLOSCompBiol
